# Descriptive molecular pharmacology of the δ opioid receptor (DOR): A computational study with structural approach

**DOI:** 10.1371/journal.pone.0304068

**Published:** 2024-07-11

**Authors:** Guillermo Goode-Romero, Laura Dominguez

**Affiliations:** Departamento de Fisicoquímica, Facultad de Química, Universidad Nacional Autónoma de México, Mexico City, Mexico; Dr Reddy’s Institute of Life Sciences, INDIA

## Abstract

This work focuses on the δ receptor (DOR), a G protein-coupled receptor (GPCR) belonging to the opioid receptor group. DOR is expressed in numerous tissues, particularly within the nervous system. Our study explores computationally the receptor’s interactions with various ligands, including opiates and opioid peptides. It elucidates how these interactions influence the δ receptor response, relevant in a wide range of health and pathological processes. Thus, our investigation aims to explore the significance of DOR as an incoming drug target for pain relief and neurodegenerative diseases and as a source for novel opioid non-narcotic analgesic alternatives. We analyze the receptor’s structural properties and interactions using Molecular Dynamics (MD) simulations and Gaussian-accelerated MD across different functional states. To thoroughly assess the primary differences in the structural and conformational ensembles across our different simulated systems, we initiated our study with 1 μs of conventional Molecular Dynamics. The strategy was chosen to encompass the full activation cycle of GPCRs, as activation processes typically occur within this microsecond range. Following the cMD, we extended our study with an additional 100 ns of Gaussian accelerated Molecular Dynamics (GaMD) to enhance the sampling of conformational states. This simulation approach allowed us to capture a comprehensive range of dynamic interactions and conformational changes that are crucial for GPCR activation as influenced by different ligands. Our study includes comparing agonist and antagonist complexes to uncover the collective patterns of their functional states, regarding activation, blocking, and inactivation of DOR, starting from experimental data. In addition, we also explored interactions between agonist and antagonist molecules from opiate and opioid classifications to establish robust structure-activity relationships. These interactions have been systematically quantified using a Quantitative Structure-Activity Relationships (QSAR) model. This research significantly contributes to our understanding of this significant pharmacological target, which is emerging as an attractive subject for drug development.

## Introduction

The δ receptor (DOR, OPRD1, DOP, or δ opioid) [1] is a member of the G protein-coupled receptor (GPCR) superfamily that binds several peptide and non-peptide ligands of both, endogenous and exogenous sources. The δ receptor is involved in multiple physiological systems and pathways, prominently related to the nervous system. Many δ receptor agonist ligands share their activity with their relatives μ (MOR) and κ (KOR) opioid receptors, which bind morphinan-core alkaloids, such as morphine, from *Papaver somniferum* (poppy) and opium, as well as other related plant narcotic sources. Thus, the morphinan-derived alkaloids are known as opiate ligands, and typically possess the activity of narcotic analgesia due to their narcosis induction. Due to the opiate pharmacological actions, the endogenous ligands of the three opioid receptors were then named opioid ligands, which are all peptides. The main opioid ligands of δ receptor are the pentapeptides enkephalin L (Tyr-Gly-Gly-Phe-Leu) and enkephalin M (Tyr-Gly-Gly-Phe-Met), discovered since 1975 [2], as well as the oligopeptides endomorphin-1 and -2, α-, β-endorphins, and others, that are also MOR and KOR agonists [3]. Several peptides from natural sources are capable of activating δ receptor, such as casomorphins from casein during milk digestion, gliadorphins from gliadin in gluten partial hydrolysis, rubiscolins from the ubiquitous enzyme RuBisCO of plants, and deltorphins from the skin poisonous secretion ‘*kambo*’ from *Phyllomedusa* frogs [4–18].

The landscape of opioid pharmacology is currently witnessing a paradigm shift towards δ and κ receptors [19–23], due to their potential to provide pain relief with decreased liability for abuse and dependency. These receptors have unique interactions with their ligands that not only mediate analgesic effects but also show promise in modulating mood disorders and neuroprotective effects without the profound addictive qualities associated with MOR agonists. The δ receptor became a relevant drug target since it has been described as an alternative of MOR agonists as severe pain reliever [24]. Targeting δ and μ receptors has shown synergistic analgesic effects and diminished tolerance and dependence [25] in comparison to targeting μ receptor solely. Further, DOR has been implicated in gastrointestinal functions and immune modulation and as an important effector in the central nervous system (CNS), since it is reported that the agonists decrease the release of proinflammatory cytokines in models of induced colitis [26]. The δ receptor signaling has also been implicated in everyday cognition, learning, memory, and gratification [27, 28], and in pathological processes such as some types of depression and anxiety [24], drug dependence, cognitive impairment, and part of key processes in neurodegenerative entities such as Alzheimer’s disease [29] and amyotrophic lateral sclerosis, among others. Evidence suggests that at the early stages of Alzheimer’s disease, there is a marked change in opioid signaling, characterized by a depletion of opioid peptides and an increase of enkephalins. This alteration in signaling dynamics leads to a decreased expression of the δ opioid receptor particularly in areas associated with learning and memory, such as the entorhinal cortex and the hippocampus [30, 31] as well as regions involved in emotional processing, such as the amygdala [32]. These changes are hypothesized to contribute to the cognitive and behavioral symptoms observed in Alzheimer’s disease, making the δ receptor a potential target for therapeutic intervention. In animal models, the modulation of δ by agonists, partial agonists, antagonists, and inverse agonists led to notable effects on neuronal and cognitive functions. Among them, the inactivation of δ signaling via inverse agonists results in short-time stress reduction, while the δ agonists induce learning memory impairment [33]. The δ receptor also influences cocaine and alcohol addiction since the antagonists prevent cocaine-seeking behavior, with the evidence of proenkephalin depletion [34] and the evidence of the contrasting effects of the DOR dimers, the heterodimer δ_1_ and the homodimer δ_2_ on alcohol intake [35].

Moreover, it is known that the opioid crisis generated by the abuse of classical μ agonists like diamorphine (heroin), desomorphine (namely, the main constituent of ‘*krokodil’*), and fentanyl gave rise in part to research of alternative opioid profiles of both, non-morphinan scaffold and lesser affinity to μ receptors [24, 36]. These facts made the δ-selective ligands a promising approach to the opioid system, although cautiously knowing the inherent risks of the DOR full activation, as it carries the misuse of deltorphins contained in the exotic ‘Kambo’ preparations consumed for recreational purposes, such as tachycardia, vomiting, convulsions, transient syndrome of inappropriate antidiuretic hormone secretion, and even rhabdomyolysis [37]. The elucidation of the DOR activation mechanisms enhances our understanding of key structure-activity relationships (SAR), particularly concerning δ-targeting drugs. These drugs potentially represent a safer class of analgesics with less abuse potential compared to traditional μ-opioid receptor agonists. By shifting the focus to δ-targeting drugs, we aim to contribute to the development of pain management strategies that mitigate the risk of addiction and other serious side effects associated with opioid misuse. The selective δ-targeting benzhydrylpiperazine scaffolds, discovered with the high-selective δ agonist BW373U86 [38], constitute a novel lead in the investigation for selective agents, and many of them possess anxiolytic and antidepressant effects *in vivo* [27, 39, 40]. Nevertheless, some compounds of the series are known to be causative of seizures [39, 41]. This fact motivated the search for non-convulsive, selective δ agonists that led to the agonist DPI287, the lesser convulsion-inducer from its group, where SAR play an important role.

Nowadays, there is a few experimental evidence at molecular level with DOR, but a wide description of Class A GPCRs become available, and thus, it is feasible correlate the data. In addition to our study’s contributions, we acknowledge the broader landscape of computational methods focusing on MD and Structure-Activity relationships (SAR), among others [42–50] that play a pivotal role in the drug design process. In our study, the focus on MD and SAR is built upon a foundation of *in silico* techniques that have been instrumental in advancing our understanding of GPCR-ligand interactions. These methodologies, as detailed in the referenced studies, enable the analysis of the dynamic interplay between DOR and various ligands. The insights gleaned from such studies are crucial to our investigation, informing the design and interpretation of our MD simulations and aiding in the elucidation of SARs critical for the development of targeted therapies. Our research question cares about the characterization of conformational ensembles of delta receptor, as a response to its interactions with ligands with specific and representative functional activities. In this work, we employed conventional Molecular Dynamics (cMD) and Gaussian-accelerated Molecular Dynamics (GaMD) simulations to describe the structural properties and key interactions of seven δ receptor systems in different functional states: the apo-receptor and the complexes interacting each one, with two antagonists, one partial agonist, two full agonists, and one inverse agonist. GaMD was selected because this method adds a boost to the potential energy, enhancing access to certain conformational features that require sorting out high energy barriers relevant to the conformational changes that characterize the active states. Such enriched sampling is particularly crucial in elucidating the dynamic processes of GPCR activation and the impact of various ligands on these states. Additionally, we analyze four full agonists, a biased agonist, three antagonists, and other inverse agonist to compare the conformer ensembles, ligand interactions, and functional findings. Our study employs molecular dynamics simulations to gain a detailed understanding of how the different ligands interact with the δ-opioid receptor system and what characteristics influence their functional activities. The significance of our research is demonstrated by the detailed insights we have obtained on the receptor-ligand binding mechanisms. These findings are crucial for the development of targeted therapeutics and provide a basis for future experimental validation.

## Material and methods

**Fig 1:**
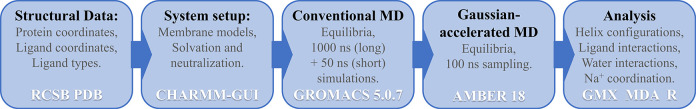
General methodology of our work. The details are described in the following sections.

The general depiction of the methodology is shown in Fig 1.

**Fig 2:**
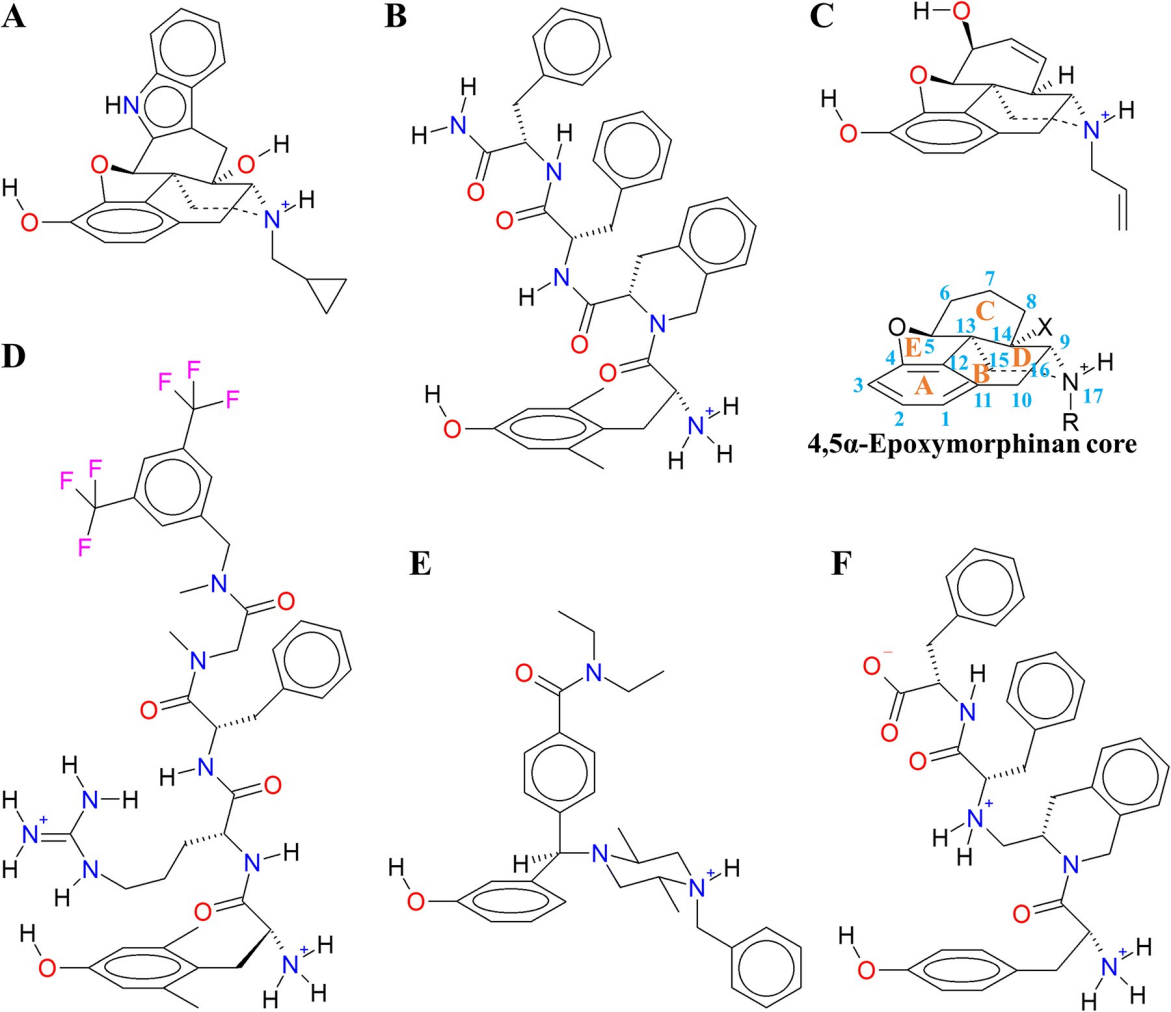
Chemical structure of the main DOR ligands in our study. **(**A) Naltrindole (NLT, a δ_2_-selective morphinan antagonist), (B) DIPP-NH_2_ (a non-selective peptide δ-antagonist), (C) nalorphine (NLR, δ-non-selective, morphinan partial agonist), (D) KGCHM07 (bifunctional, δ-selective, peptide agonist), (E) DPI287 (δ-selective, benzhydrylpiperazine class agonist), and (F) TIPPψ (δ-selective, pseudopeptide inverse agonist).

Structural data. We study the seven δ monomer systems with cMD and GaMD simulations. The structural data were taken from the Protein Data Bank (PDB) [51], for the apo-receptor, and the complexes with the following ligands: The morphinan, selective antagonist naltrindole [52, 53], the μ-agonist/δ-antagonist tetrapeptide DIPP-NH_2_ (2,6-Dimethyl-*L*-tyrosinyl-(*3S*)-1,2,3,4-tetrahydroisoquinoline-3-carbonyl-*L*-phenylalanyl-*L*-phenyla-laninamide; Dmt^1^-Tic^2^-Phe^3^-Phe^4^ -NH_2_) [54], the partial agonist nalorphine (modeled from the naltrindole complex), the peptidomimetic, bifunctional NK_1_ (neurokinin 1)/δ-agonist KGCHM07 (*N*-(*bis*(3,5-trifluoromethyl)benzyl)-*N*-methyl-2,6-dimethyl-*L*-tyrosinyl-*D*-argi-nyl-*L*-phenylalanylsarcosinamide; Dmt^1^-*D*-Arg^2^-Phe^3^-Sar^4^ -N(CH_3_)(Bz(CF_3_)_2_)) [24], the benzhydrylpiperazine agonist DPI287 [24], and the complex with the DIPP-NH_2_-structurally related, inverse agonist pseudopeptide TIPPψ (*L*-Tyrosinyl-(*3S*)-1,2,3,4-tetrahydroisoquinoline-3-methylene-*L*-phenylalanyl-*L*-phenylalanine; H_2_^+^-Tyr-Tic(CH_2_NH_2_^+^)-Phe-Phe-O^-^) [55] (Table 1 and Fig 2). The additional systems that we studied, with the inverse agonist SYK657 [33], the antagonist naloxone, buprenorphine, Compound 4 [56], the biased agonist PN6047 [57], the agonists morphine, BW373U86 [38], deltorphin II, and enkephalin L, were described in the S1 Table and S1 Fig of the Supporting Information. The ligand information was taken from IUPHAR and PubChem [1, 58]. To assess the main differences among the structural and conformational ensembles of each system, we carried out 1 μs of cMD, and subsequent 100 ns of GaMD as we describe below, since the activation of GPCRs is reported rounding a time scale of 1 μs.

**Table 1:** General description of our simulated δ systems. The simulation lengths refer to cMD and GaMD. All the systems were simulated 1 μs as cMD with GROMACS. Then, two replicates of 50 ns as cMD, and finally, 100 ns of GaMD with AMBER18.

System	Ligand name	Ligand structural class	Ligand activity	PDB ID template
Apo-δ	(*Apo*)	(*none*)	(*none*)	4EJ4 [52], 4N6H [53]
δ-NLT	Naltrindole	Morphinan	δ_2_-Sub-selective antagonist [91]	
δ-DIPP	DIPP-NH_2_	Peptide	Non-selective: δ antagonist/μ agonist	4RWA, 4RWD [54]
δ-NLR	Nalorphine	Morphinan	Non-selective partial agonist	4EJ4, 4N6H
δ-KGCH	KGCHM07	Peptide	Bifunctional: δ agonist/NK1 antagonist	6PT2 [24]
δ-DPI	DPI287	Benzhydrylpiperazine	Selective agonist	6PT3 [24]
δ-TIPP	TIPPψ	Pseudopeptide	Selective inverse agonist	4RWA, 4RWD, 6PT2

System setup. The apo-δ and δ-ligand complexes were modeled according to the available information in UniProt: P41143 [59], with *N*-(2-desoxy-2-amino-β-D-glucopyranosyl)-acetamide (N-acetylglucosamine, NAG) moieties in the residues N18 and N33 of the N-terminus, as well as a *S*-palmitoylation in the residue C333 (C^Palm^) located in the juxtamembrane helix 8. The OPM (Orientation of Proteins in Membranes) database has been instrumental in our study for the accurate positioning of receptor systems within lipid bilayers. This tool employs an algorithm that minimizes the water-lipid transfer energy, enabling precise estimation of the embedded protein’s orientation by considering membrane depth, the protein’s center of geometry, and angular orientation. Such orientation is crucial for realistic simulations of membrane proteins, as it ensures the physiological relevance of the model structure [60]), and CHARMM-GUI [61, 62] servers, respectively. The membrane composition was constituted by 1-palmitoyl-2-oleoyl-*sn*-glycero-3-phosphorylcholine (POPC), and the structural cholesterol present in PDB entries. We used the TIP3P water model [63], and a neutral concentration of 0.15 M of sodium chloride. All the ligands were parameterized using CHARMM-GUI server, with CHARMM36m forcefield, from the initial coordinates, and the ligand parameterization from a *mol2* format file.

Conventional Molecular Dynamics (cMD). We carried out the cMD simulations with GROMACS 5.0.7 [64, 65], with the CHARMM36m forcefield, as other previous GPCR studies [44, 66], and LINCS [67] algorithm to constraint bonds involving hydrogen atoms. Initially, we performed an energy minimization with the Steepest Descent algorithm, and then, two isomolar-isochoric-isothermal (NVT) and four isomolar-isobaric-isothermal (NPT) equilibria, with decreasing restrictions to the backbone, sidechains, ligand heavy atoms, and one dihedral angle of POPC. The reference temperature for NVT and initial velocity generation was at 310 K, with the Berendsen thermostat [68], a time constant of 1.0 ps^-1^, and a timestep of 0.001 ps. We choose the temperature of 310 K to emulate human, physiological conditions. For the NPT equilibria, we fixed the reference pressure by 1 bar in a semi-isotropic ensemble, with Berendsen barostat, with a time constant of 5.0 bar^-1^, and an isothermal compressibility of the solvent of 4.5×10^−4^ bar^-1^, and a timestep of 0.001 ps for the first two equilibria, and 0.002 ps for the rest. Then, the MD production was performed without restrictions, NPT ensembles without velocity generation for 1 μs (from here, *long cMD* relative to the next step), with velocity rescale thermostat [69] and Parrinello-Rahman barostat [70], and a timestep of 0.002 ps. The thermostat algorithm decreases exponentially the temperature fluctuations with respect to the target temperature value.

Gaussian Accelerated Molecular Dynamics (GaMD). GaMD is an enhanced sampling technique that accelerates the conformational sampling of the systems beyond what is achievable with conventional MD. This accelerated sampling methodology adds a boost to the potential energy, to favor the access to certain conformational features that require sorting out high energy barriers. Our decision to utilize GaMD was based on its advanced capability to enhance conformational sampling efficiency. GaMD has been shown to better preserve the integrity of protein structures while still accelerating the sampling of relevant conformational states. This is critical for GPCR systems where the accurate representation of transmembrane regions is essential for understanding ligand interactions and receptor activation. We obtained the final configurations and used the AMBER18 packages to build the accelerated sampling and initial coordinates. We transformed the input configurations with charmmlipid2amber script and ANTECHAMBER [71] to parameterize the C333^Palm^ residue and tLEaP with Protein.ff14SB [72], GAFF2 [73], Lipid14 [74] and GLYCAM_06j-1 [75] forcefields. We simulated two NVT and four NPT equilibria with decreasing restraints, the SHAKE [76] algorithm for constraining bonds with hydrogen atoms, and a reference temperature of 310 K, and then 1 bar as reference pressure with the Monte Carlo barostat [77]. First, we simulated 50 ns of NPT cMD (from here, referred to as *short cMD*, relative to the cMD of 1 μs), and finally, 100 ns of NVT GaMD [78], with a dual boost to dihedral and total energy, and Gaussian σ_0;P_ and σ_0;dih_ parameters of 6.0 kcal/mol (the Gaussian standard deviation parameters σ were selected according to the studies of J.A. McCammon and cols. [78], which suggest values rounding 10k_B_T). The dual boost is applied to the total and dihedral energy terms, with the aim to favor access to previously high-barrier-energy states. We carried out two replicates of the GaMD simulations.

Analysis. We used GROMACS 5.0.7 programs to compute the root of mean squared deviation (RMSD; fitted to the backbone transmembrane domain TMD with respect to the initial conformation), the root of mean square fluctuation (RMSF; with respect to the average conformation), with the aim to analyze the average displacements in the simulations; Gromos clustering [79] with elbow rule [80] (to obtain an equilateral hyperbole-shaped profile of the cluster sizes) to find the representative, conformational configuration of the receptor in each system, secondary structure profiles to examine the folding or unfolding, matrices of contacts to characterize the interactions, minimal distance and dihedral calculations to evaluate the contacts and torsions that may be relevant; MDAnalysis [81] for water molecules counting and frequency of contacts, R [82, 83] for the plotting of RMSD distributions, Gnuplot for plots [84], and PyMOL [85], VMD [86], and ISIS Draw [87] for figures. We converted the AMBER outputs to GROMACS files with cpptraj [88].

Additionally, we performed a Quantitative Structure-Activity Relationship (QSAR) analysis for several DOR ligands with R. We optimized the geometry of the molecules with Gaussian16 (M06/6-311+G(2d,p)) [89, 90].

## Results and discussion

We discuss our findings of the δ receptor in the following sections.

### I. Conformational changes of DOR at receptor scale: TM1-TM2, TM5-TM6, and TM7 are key indicators of the functional state

**Fig 3:**
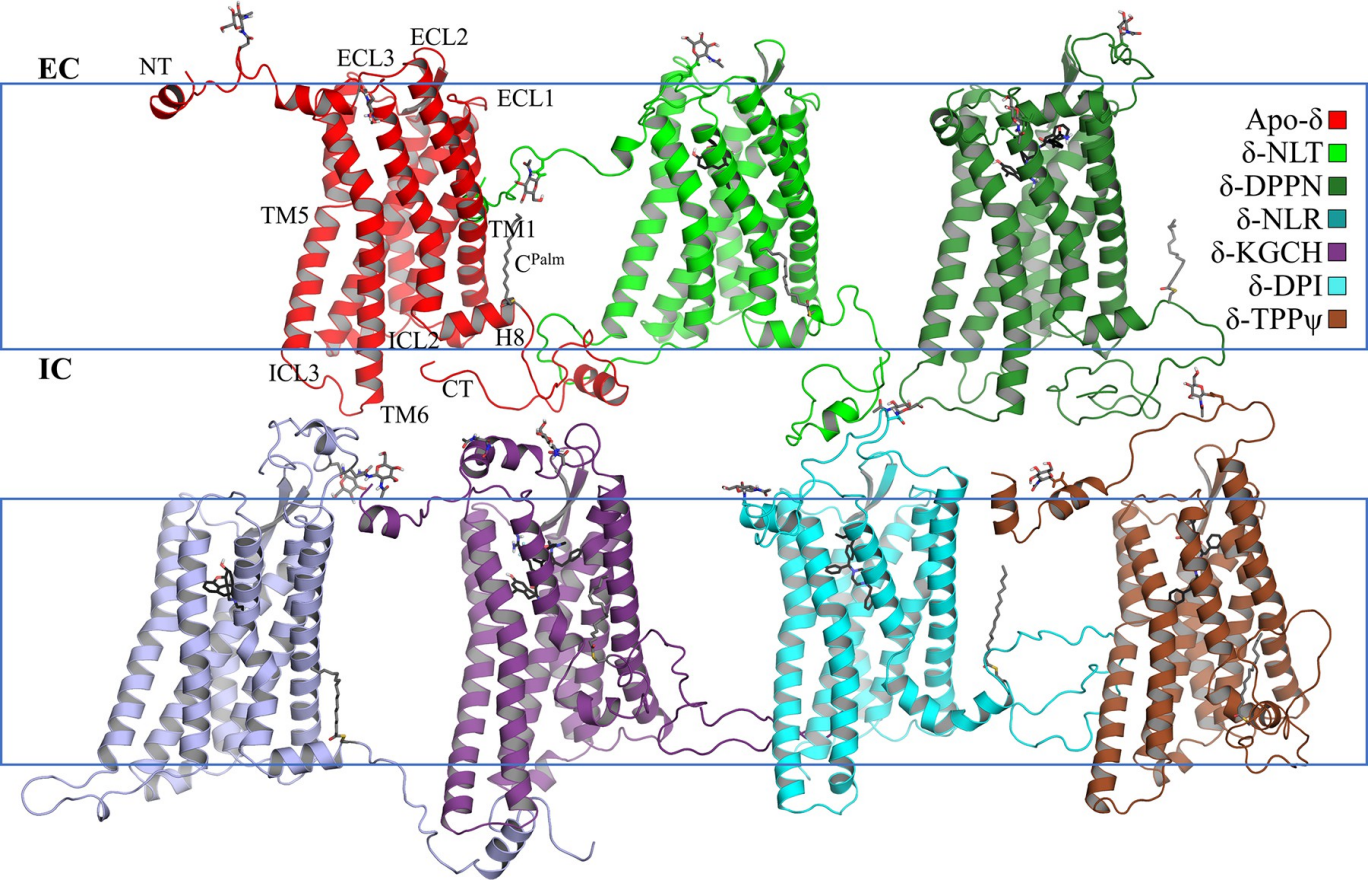
Representative conformers of our seven main simulated DOR systems: Apo-δ, δ-naltrindole, δ-DIPP-NH_2_, δ-nalorphine, δ-KGCHM07, δ-DPI287, and δ-TIPPψ. In the apo, naltrindole, DIPP-NH_2_ and nalorphine complexes, TM5 and TM6 remain distant between them, whilst in the KGCHM07, DPI287, and TIPPψ systems, they are positioned closer to each other at the IC side. H8 experienced unfolding or embedding deeper in the membrane and a large torsion.

Our DOR simulations found relevant conformational differences induced by the activity of its bonded ligand: naltrindole (NLT) and DIPP-NH_2_ as antagonists, and nalorphine (NLR), whereas DPI287 and KGCHM07 as agonists (Fig 3). Conformational differences in our agonist and antagonist simulated systems are related to their individual variations and are consistent in our GaMD simulations.

Since the δ receptor possesses constitutive activity, the inverse agonist-induced state can be remarked by the difference between an antagonized state and such inactivated state. It has been reported experimentally that naltrindole possesses very low intrinsic efficacy activating DOR, about 7.5% [33, 92], practically acting as a neutral antagonist, whilst TIPPψ has negative intrinsic efficacy. Due to their neutral influence on the experimental activity, we refer as *stative* (in analogy to a ‘static state’) the functional states led by antagonist ligands, and *inactive*, the functional states induced by inverse agonists, where the experimental activity of the receptor decreases further than the expected level in the absence of any activators (negative efficacy). In our study, we differentiate between active, inactive, and partially active δ-opioid receptor systems by employing two principal approaches. The first involves assessing the ligands’ functional activity through experimental data, determining whether they act as agonists, antagonists, or inverse agonists. The second approach synthesizes structural insights from our molecular dynamics simulations, corroborated by established findings in GPCR research. The significance of identifying stative and partially active systems lies in their potential therapeutic applications. These states are critical for designing drugs that can selectively activate beneficial signaling pathways while minimizing those that lead to adverse effects.

Our classification is reinforced by observed features common across GPCR studies [93–103], such as the TM5-TM6 helical configuration, the presence of the transmission switch at TM6, the pattern of ionic lock residues, and the role of water molecules within the interhelix pore. These structural markers are indicative of the receptor’s functional state and, thus, are integral to our classification strategy, such as TM5-TM6 configuration, transmission switch located at TM6, the configuration of the ionic lock residues in the intracellular side of the receptor, the water molecule presence within the interhelix pore, among others.

#### a) Stative and partial active systems

Those systems (NLT, DIPP-NH_2_ and NLR complexes) reach dynamic equilibrium states at around 0.6 and 0.9 μs of the long cMD of 1 μs, interpreted from the RMSD profiles of the TMD backbone (S2 Fig). As expected, we only observed small conformational changes since the antagonized complexes do not experience functional state changes in the presence of the blocker ligands. There is an exception in our additional systems with the antagonists, buprenorphine and naloxone, that we explain below.

A common conformational feature in the non-agonized apo, NLT, DIPP-NH_2_, and NLR systems is the separation between the intracellular (IC) ends of transmembrane helix 5 (TM5) and 6 (TM6) with an extended and unfolded conformation of the intracellular loop 3 (ICL3), the latter except in apo-δ. In Class A GPCRs, the separation of IC end of both helices is related with a lack of activation. Also, an inward inclination of the extracellular (EC) end of TM1 almost reaches perpendicular conformations with respect to the bilayer. The transmembrane helix (H8) retains its folding and with a relatively invariant inclination. The cavity at the EC side of the receptor remains open, except in apo-δ (S3 Fig). The *N*-acetylglucosamine moiety bound to the N18 sidechain is embedded in the POPC-head (phosphates) and -neck (glycerol ester) layers of the membrane only in the two antagonized systems and not in the active nor the inactive systems.

#### b) Active and inactive systems

TMD backbone RMSD profiles in the active systems do not stay in equivalent, dynamical conformations. The KGCHM07, DPI287, and TIPPψ systems, although possessing opposite functional states, share several conformational similarities. This is explained since inverse agonists promote the dissociation of the pre-coupled G protein to the receptor [104], while agonists stabilize the coupling with G proteins.

**Fig 4:**
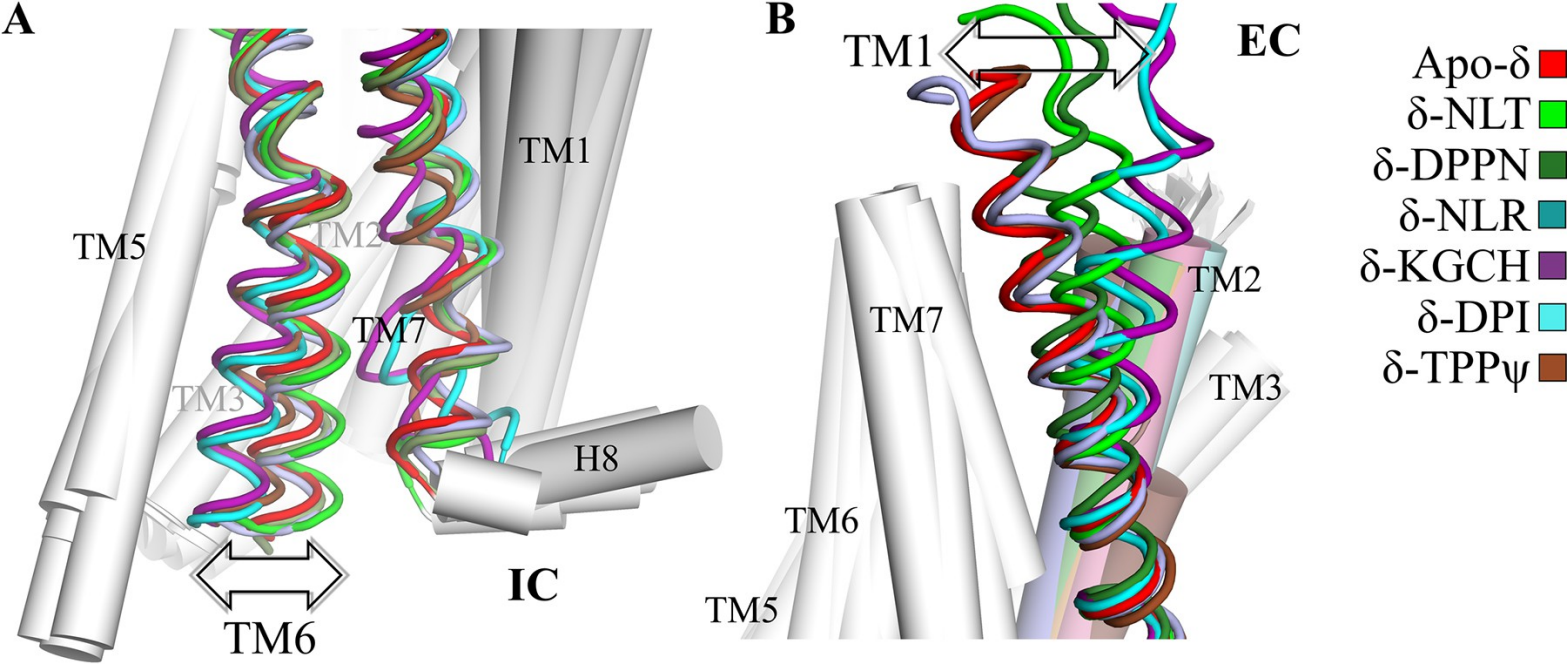
Contact patterns between pairs of TM helices in the seven systems. (A) The intracellular ends of TM6 and TM7, and (B) the extracellular ends of TM1 and TM2. The active and inactive systems have a lesser number of contacts between TM6 and TM7, while have more contacts between TM1 and TM2.

TM5 and TM6 remain closer to each other at their IC end, and ICL3 folds helically into them in the active systems, and only to TM5 in the inactive complex (Fig 4). The concerted separation of the IC ends of both helices from the helix bundle, is related with an incipient activation, since TM6 and TM7 decreases their interactions, and the IC side of the receptor become available to posterior interactions with incoming transducers as proteins G. H8 experiences abrupt changes in folding and torsions: in the active systems, H8 tends to embed into the bulk of the membrane, while in the inactive complex, it tends to move far away from TM1 (S3 Fig). During our long cMD simulations of the active and inactive systems, TM7 exhibits a partial unfolding that is absent in the stative systems (Fig 4 and S4 Fig). This feature is related to H8 changes in those systems (S5 Fig), as it is reported during the activation [105]. The kink where helicity breaks is at the residues L313^7.48^ and N314^7.49^ (we use the Ballesteros-Weinstein nomenclature [106]), where the latter is known to participate in the sodium cation coordination. The importance of N314^7.49^ highlights since the mutation of this residue, along two adjacent ones, lead to transform the activity of the antagonist naltrindole, to a β-arrestin-biasing [53].

The two peptide complexes (KGCHM07 and TIPPψ) tend to decrease the EC side area and displace whole TM1 towards the EC and TM5-6 into the IC regions, whilst the DPI287 complex roughly keeps its geometrical shape. TM1 and TM2 are indeed integral to the orthosteric site at their EC termini, harboring key amino acid residues such as Q105^2.60^ and Y109^2.64^. These residues are pivotal for conformational changes that correlate with different functional states of the receptor, as we discuss below. The findings described between both types of agonists in our simulated systems are consistent with the experimental observations reported for these systems [24], which led us to remark differences in the mechanism of these ligands, or an affinity dependent of the distinct protomers.

### II. The ligand interactions: The peptides establish distinctive interactions in which a bulky group determines its selectivity

**Fig 5:**
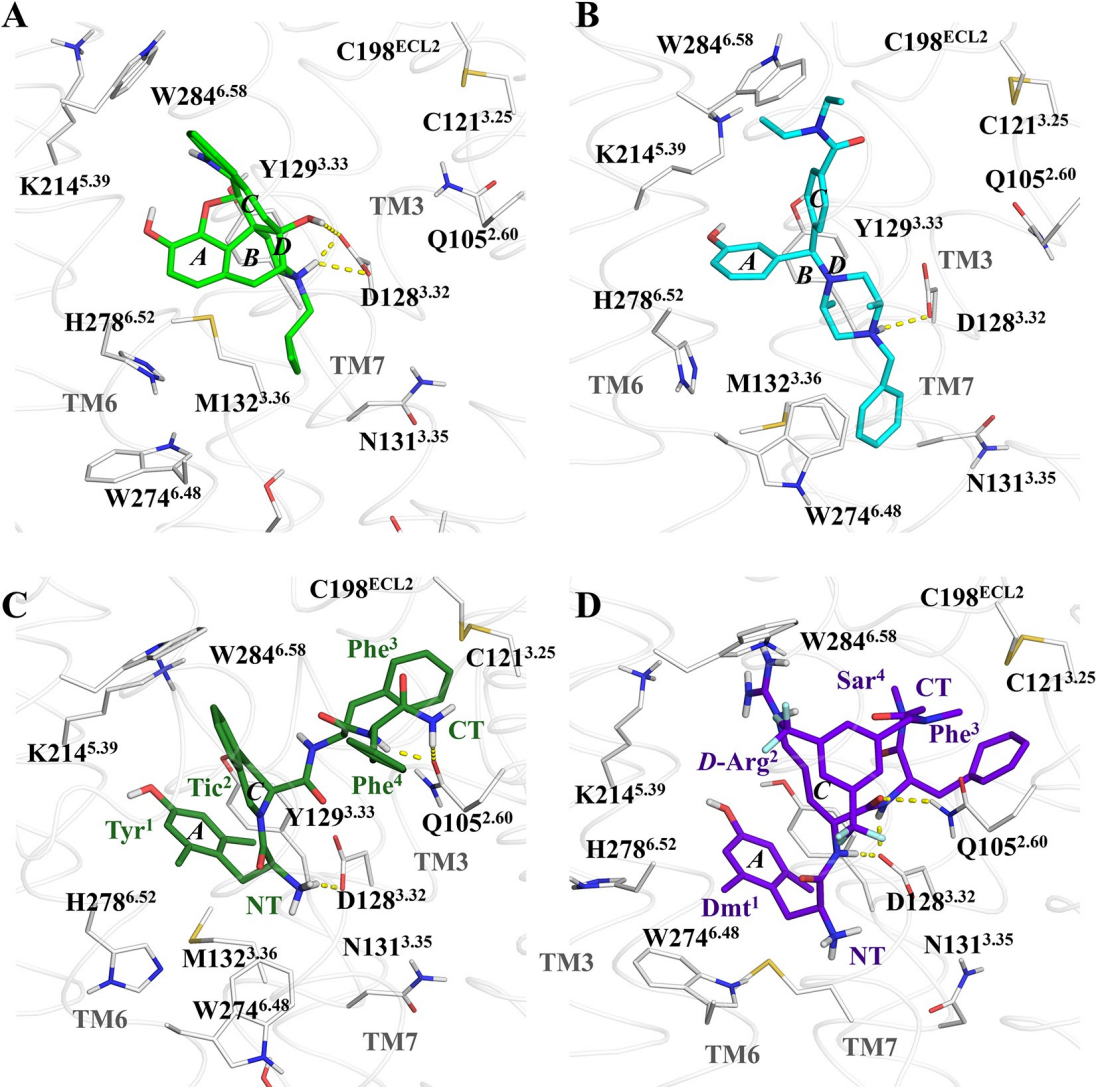
Relevant contacts between the ligands in the six complex systems. (A) The morphinan antagonist naltrindole, (B) the benzhydrylpiperazine agonist DPI287, (C) the pseudopeptide, inverse agonist TIPPψ, and (D) the peptide, full agonist KCGHM07. The uppercase letter *A*-*D* identifiers in the snapshots correspond with the rings in the morphinan skeleton. D128^3.32^ is the anionic counterpart in the mostly conserved -but not exclusive- salt bridge formed with the ligand within the orthosteric site (OSS). Y129^3.33^ interacts with the phenol function of ring A of the morphinans, tyrosine or dimethyltyrosine (Dmt) moieties; and with the hydrogen-bond-donor in ring C, when the ligand possesses it, as well as K214,^5.39^ by its hydrophobic and cation parts. H278^6.52^ also interacts with the phenol group through a water molecule or directly. The transmission switch W274^6.48^ is in contact with either, the ring A, or the *N*^17^-attached group (*N*-substituent) in the ligand. Q105^2.60^ interact with the peptide bonds of the peptide bounded agonist ligands, and through hydrophobic contacts with their Phe residues. It is evident that the *N*-benzyl group of DPI287 tends to interact with N131^3.35^, and the Tyr^1^ residue (ring A) in TIPPψ rotates through W247^6.48^ to a greater extent than the other ligands.

We found that the class and function of the ligands are closely related to structural changes of the receptor. We first analyzed the ligand class findings (Fig 5) and then moved on to examine the interactions with the receptor, based on the structure of the morphinan core (*i*.*e*., the scaffold of the opiates) for consensus and therefore, the structure of naltrindole, nalorphine, and the additional studied systems: naloxone, buprenorphine, SYK657 (all of them bearing an additional 14-hydroxyl group), and morphine; that we detailed them in a bin-colored matrix (S6 Fig) to remark the overall similarities.

**Fig 6:**
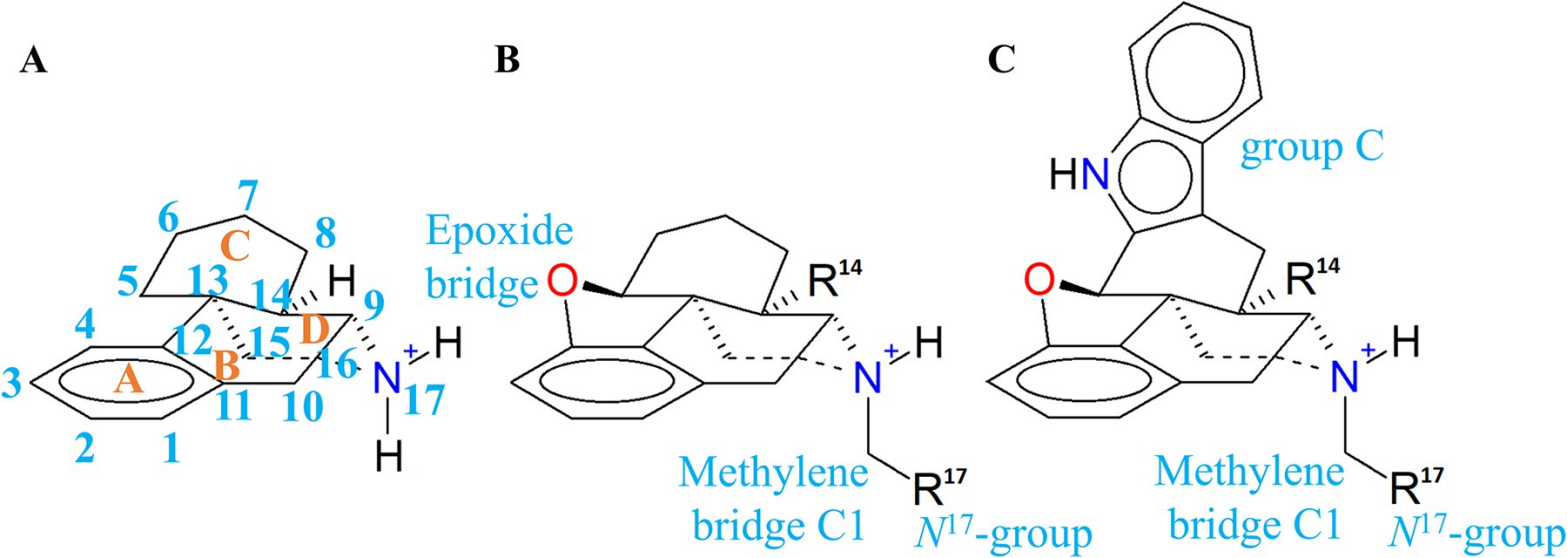
Morphinan scaffold structures. (A) Morphinan, (B) 4,5-epoxymorphinan, and (C) morphindole.

The morphinan core (Fig 6) that we used as a reference scaffold possesses three key components **(1)** a phenol function that is denominated as ring A, **(2)** a tertiary ammonium group (N^17^) linked to rings B/D, **(3)** an unsaturated or bulky group (C-group) at ring C, and **(4)** only for the alkaloids, an epoxide bridge denominated ring E. For clarity, we refer to the ligand residues in the three-letter code and the receptor residues in the one-letter code.

### Ring A: The phenol function that forms water bridges with Y129^3.33^ and H278^6.52^

The phenol function of the ligands (ring A of the morphinans, residue 1 of the peptides, and the *meta*-substituted ring of the benzhydryl moiety; see Fig 1 and S1 Fig) form hydrogen bonds with at least one water molecule, which in turn forms another hydrogen bond with the sidechain of either Y129^3.33^ and H278^6.52^, in agreement with the experimental structures [52]. Interestingly, Y129^3.33^ also interacts with the ether oxygen of the morphinan ring E. The phenol function is a conserved feature in many of the opioid ligands and is present in all our studied ligand systems. It is separated by two carbons from a positive charge that mimics the N-terminal Tyr in endogenous peptides [52], or Dmt of the exogenous. The phenol is a pharmacophore very common for ligand binding to opioid receptors, and even, the presence of a N-terminal tyrosine residue in certain peptides let them to interact with DOR in a variable fashion, as it is reported for some somatostatin analogues [107], as Compound 4. The lack of the hydroxyl group, replaced by the methoxyl group in codeine (*O*^3^-methylmorphine), results in a very weak ligand affinity for the opioid receptor in comparison with morphine. Nevertheless, the hydroxyl-lacking ligands such as SNC80 (*O*-methyl-BW373U86) [34], SNC162 (deoxy-BW373U86) [50], PN6047 [57] (see S1 Fig), as well as other ligands with different binding pose like fentanyl [108] and its relatives, are capable to bind and activate DOR in a quasi-selective or selective fashion. Samidorphan [95, 109] exerts antagonistic activity in DOR (with 21% of efficacy with respect SNC80, settled to 100%) [110] despite the shift of the hydroxyl to a carboxamide group. In our PN6047 simulated system, which lacks the phenol but has a carboxamide group like samidorphan, the bulky group guides the receptor-ligand interaction, as discussed in the next sections and suggested in the literature [24].;

### Rings B and D: The 14-hydroxyl and tertiary ammonium groups interact differentially with D128^3.32^

The protonated amino group of our different ligand complexes, interacts through a saline bridge with D128^3.32^, with particularities in each complex. The agonistic benzhydrylpiperazines DPI287, BW373U86, the biased benzhydrylidenepiperidine PN6047, and morphine interact solely through the N^17^ atom, and the peptides form mainchain interactions, including the protonated -NH_3_^+^ of the N-terminus. The morphinans naltrindole, naloxone, buprenorphine, and SYK657, form an additional interaction through its 14-hydroxyl group along the protonated nitrogen atom and Y129^3.33^ (S7 Fig).

The group attached to the 17-position (namely *N*^17^) of the bounded ligand has been shown to influence the functionality in the receptor. The *N*^17^-cyclopropylmethyl substituent in the antagonist naltrindole possesses an intrinsic torsional contribution, confined to the three-membered ring, compared to the allylic nitrogen atom of the partial agonist nalorphine. This difference affects the adjacent methylene bridge C1 and the N^17^ atom distinctly. The allyl group is acyclic and unsaturated, and it does not experience resonance structures carrying the electrophile-like allylic C1 atom. Consequently, the increased electron-attracting character of the nalorphine N^17^ leads to an increased acidity of the group and, thus, a lesser anionic character of D128^3.32^. Moreover, the absence of a 14-hydroxyl group in nalorphine results in a distinctive ionic interaction between N^17^ and D128^3.32^. In contrast, the antagonist naloxone (with 8% to 10% efficacy [111]) carries the same *N*^17^-allyl group but possesses the 14-hydroxyl group, which increases the basicity of N^17^, in comparison with nalorphine (experimental pKa values of naloxone and nalorphine are 7.9 and 7.6, respectively [112]). Thus, nalorphine might have a more electron-deficient character on the methylene bridge compared to naloxone. The inverse agonist SYK657, which features an *N*^17^-benzyl group and the14-hydroxyl group, interacts similarly to the antagonist naloxone at this level. However, the benzyl group appears to contribute to the reduction of the basic character of D128^3.32^.

The antagonists naltrindole and buprenorphine, and the inverse agonist SYK657 share the same binding pose. Due to the torsion restrictions inherent to the whole morphinan core, the ligands remain nearly immovable, featuring the common phenolic ring A and N^17^ interactions (excepting naloxone). In contrast to the other *N*^17^-arylmethylene partners, the *N*^17^-benzyl group of SYK657 (which possesses intrinsic aromatic delocalization), does not notably contact N131^3.35^ (as mentioned above, implicated in the biased activation to the β-arrestin pathway), and it is positioned similarly to the *N*^17^-cyclopropylmethyl of naltrindole and buprenorphine. In contrast, the naloxone complex exhibits multiple differences in the ligand’s configuration, through the replicates, unlike the closely related ligand nalorphine, even in the GaMD simulations of the latter. We discuss more of the findings of the naloxone complex in S8 Fig caption. The presence of a bulkier substituent attached to N^17^ in the morphinan core than methyl has been shown to be a determinant factor for the antagonist activity, leading to significant changes in the structure-activity relationships. The introduction of electron-withdrawing groups has been explored in various cases, including the reduction of the basic property of the nitrogen atom, for instance, attaching carbonyl and sulfonyl groups, has resulted in a wide range of activities [92]. We substantiate our findings and emphasize the impact of minor structural variations on driving significant functional changes with an extensive discussion of the N^17^ groups and provide a QSAR analysis for selected δ-ligands to explore these phenomena (S19 Fig and S2–S5 Tables).

While the cationic function appeared to be essential for binding to the receptor, considering that most opiate and opioid ligands possess this characteristic, some exceptions have been reported. For instance, molecules bearing the phenol function with a non-basic amino group, are still capable of binding to the δ receptor. Examples include the carbonyl- and sulfonyl series of naltrindole derivatives, and the cyclopeptide Compound 4 (Cmp4), derived from somatostatin receptor ligands. Compound 4 lacks a protonated nitrogen atom due to its homodetic cyclopeptide nature. It forms a polar-anion interaction through Trp^2^, Tyr^3^, and Thr^4^ with D128^3.32^ (S10 Fig). While Tyr^3^ establishes the expected phenolic ring A contact, we found that Nal^1^ and Trp^2^ can engage in π-π coupling with Y56^1.39^ and Y308^7.43^. Additionally, there are hydrophobic contacts with T101^2.56^, Q105^2.60^, and K108^2.63^. In contrast, in agonist peptides it has been reported that acetylation of the N-terminus in enkephalins leads to a loss of their agonist activity and affinity to opioid receptors. Therefore, the equilibrium of various interactive contributions determines the ligand profile when direct structural variations occur.

**Fig 7:**
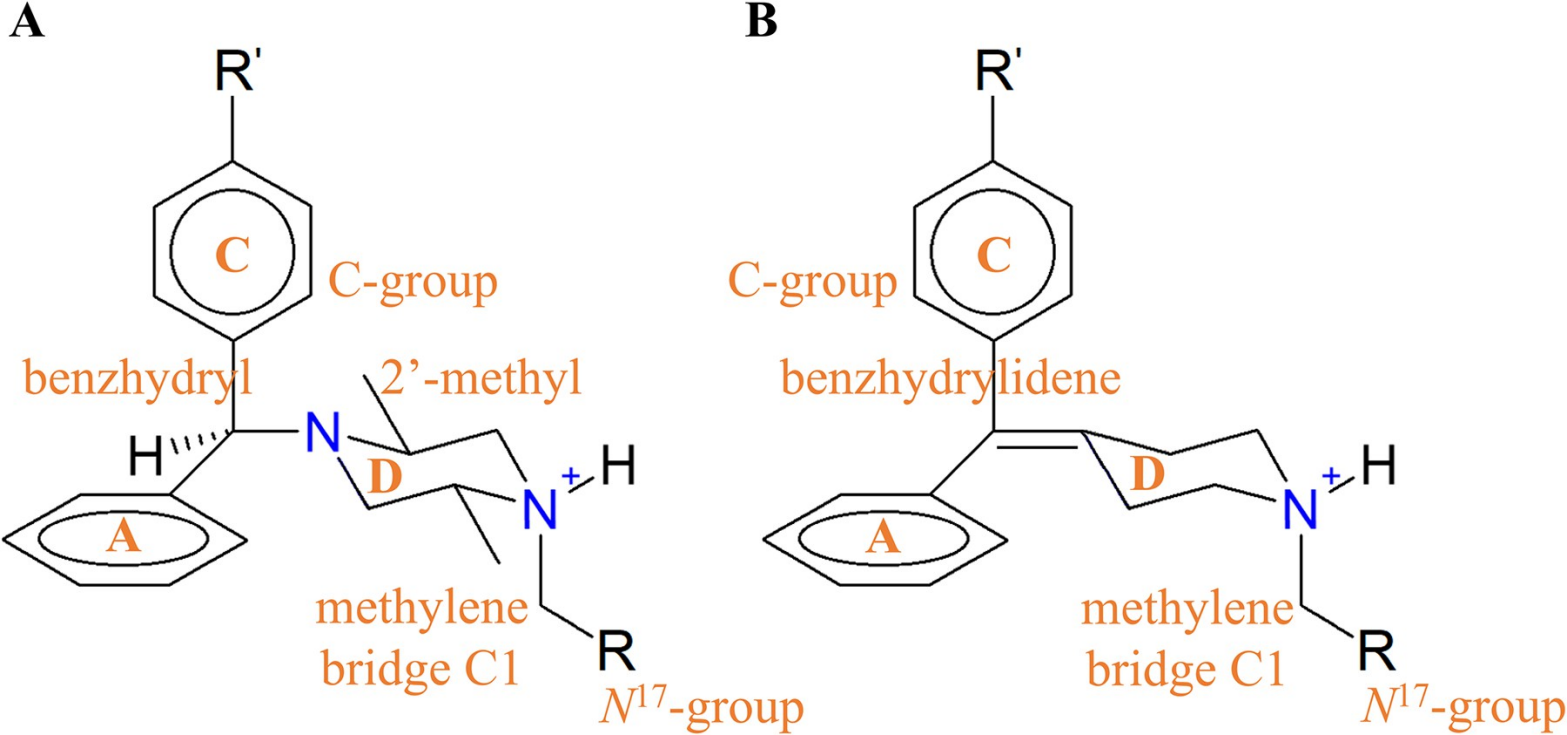
(A) Benzhydrylpiperazine core from DPI287 and BW373U86, and (B) benzhydrylidenepiperidine core from PN6047.

Regarding the agonist binding poses, DPI287, that possesses the N^17^ as a benzylic function, lacks any equivalent group to the 14-hydroxyl of the morphinans, and the shifting of its *N*^17^-benzyl to *N*^17^-allyl, as in BW373U86, which is also an agonist, in contrast with SYK657 and nalorphine, respectively. The agonists DPI287 and BW373U86 possess methyl groups on their piperazine rings (Fig 7). The 2,5-dimethylpiperazine group in those ligands may contribute substantially to the agonistic effect, since the opposite functionality observed in the *N*^17^-allyl group shared between nalorphine and naloxone, as well as the change in the functionality, from neutral to negative efficacy of the morphinans, at the shift from *N*^17^-allyl to *N*^17^-benzyl. Additionally, the N^17^ groups of DPI287 and BW373U86 exert steric hindrance on Y308^7.43^ and partially induce a kink in the helix, contributing to the unfolding of TM7 (S9 Fig), since that ligands are more flexible than morphinans.

The related biased agonist PN6047, from the benzhydrylidenepiperidine class, features an *N*^17^-(thiazole-5-yl) moiety that possesses distinct electron properties, successfully represented in the forcefield, compared to the agonist DPI287. These differences might underlie its activity. Comparing the three benzhydyl/benzhydrylidene ligands, The three ligands show several similarities in their representative poses, and in particular DPI287 and PN6047, similar interactions with the transmission switch: while the *N*^17^-benzyl and -thiazolyl groups, along with the equivalent ring A, interact with the indole sidechain of W274^6.48^, limiting the accessible rotamers of the latter, the *N*^17^-allyl group of BW373U86 does not restrict the torsional changes of the switch (S9 Fig). In fact, the switch reaches a full activation-related configuration in this system, *i*.*e*., rotated towards TM5, whilst in the DPI287 and PN6047 complexes, the rotamer is directed predominantly towards the interhelix pore. Given that it has been reported that the partial agonistic mechanism in another class A GPCR relies on the stabilization of the transmission switch through an aromatic interaction with its ligand [113], DPI287 and PN6047 apparently share a mechanistic activation pathway that could be biased (or partial) in both cases. On the other hand, BW373U86 exhibits features of a full-activation mechanism. Furthermore, the residue W174^4.50^, adjacent to Y130^3.34^ and N131^3.35^, predominates rotated towards TM3, excepting in BW373U86 complex, which rotates away the helix bundle.

Experimentally, mutation studies have demonstrated that the δ-selective antagonists, naltrindole, naltriben (the benzofuran analogue of naltrindole, and κ-(δ) oligomer agonist [114]), and naltrexone (the naloxone analogue with a shift from N^17^-allyl to N^17^-cyclopropylmethyl), bind equally well to both the wild-type δ receptor and the constitutively inactive D95N^2.50^ mutant. In contrast, the non-selective agonist bremazocine also exhibited strong interactions with the D95N^2.50^ mutant [56]. Based on these observations, it has been emphasized that the δ-selective agonists, such as DPI287, PN6047, and BW373U86, bind differently compared to the δ-selective antagonists like naltrindole, naltriben, or non-selective δ agonists like morphine and enkephalin L (ENKL). This difference may account for the distinction between nalorphine and naloxone in comparison to BW373U86, all of which possess N^17^-allyl groups.

### Ring C: The bulky and hydrophobic group interact mainly with W284^6.58^ and determines selectivity to the δ receptor

**Fig 8:**
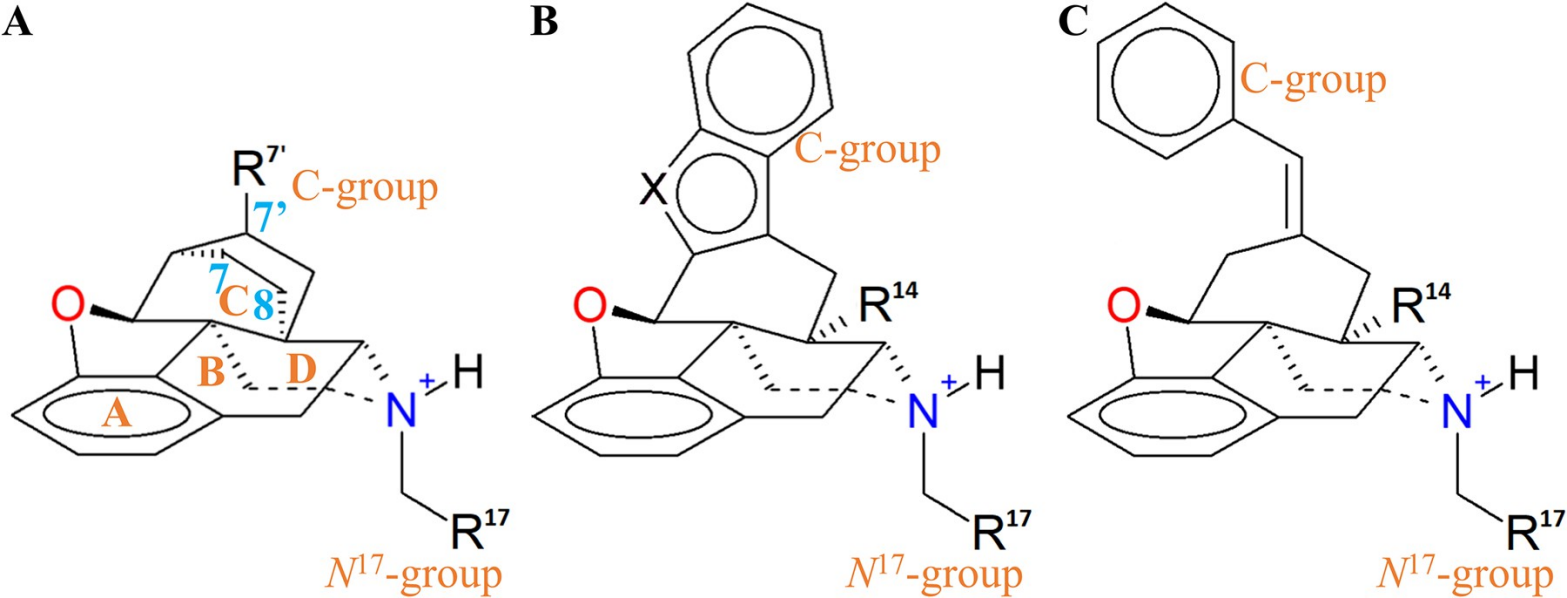
C-groups attached in the morphinan ligands. (A) In buprenorphine and other orvinols, (B) in morphindole, and morphobenzofuran ligands, such as naltrindole and SYK657, and (C) benzylidenenaltrexone and SYK656.

The substituents of the ring C of the morphinan core displays a sustained interaction with a hydrophobic cluster located at the extracellular side at TM5 and TM6, particularly, W284^6.58^ but also I277^6.51^, F280^6.54^, V281^6.55^, R291^ECL3^ (its propylene segment), and L300^7.35^. Since μ and κ receptor possess R^6.58^ and E^6.58^ at the DOR-tryptophan position, this residue plays a determinative role in the selectivity to δ. The ring C-attached groups are those at morphinan positions 6 and 7, and the bulkier and hydrophobic, the most interacting with W284^6.58^. The indole fused ring in the antagonist naltrindole, and the benzofuran in the inverse agonist SYK657 (Fig 8), function as those bulky C-groups that in turn, interact through π interactions with the mentioned site. Considering the naltrindole structure as morphindole template, the change of the indole system per quinoline (to morphoquinoline), converts it from a quasi-neutral antagonist to a partial agonist (32.4% of efficacy with respect the reference the full agonist DPDPE), whereas the shifting of indole per benzofuran (naltriben), changes the quasi-neutral effect to a very slight inverse agonist (Those ligands are selective to the δ_2_, homodimer protomer). In the case of a non-cyclic shift, such as from benzofuran to 7-benzylidene as in benzylidenenaltrexone, the inverse agonistic activity increases slightly (-10.2% of efficacy) [33, 115]. With these reported activities, it is expected that the shift of the 17-cyclopropylmethyl to 17-benzyl, produces the corresponding inverse agonists SYK657 and SYK656 (a δ_1_ inverse agonist), where interestingly the tendency preserves (-99% and -103% of efficacy, respectively) [33].

**Fig 9:**
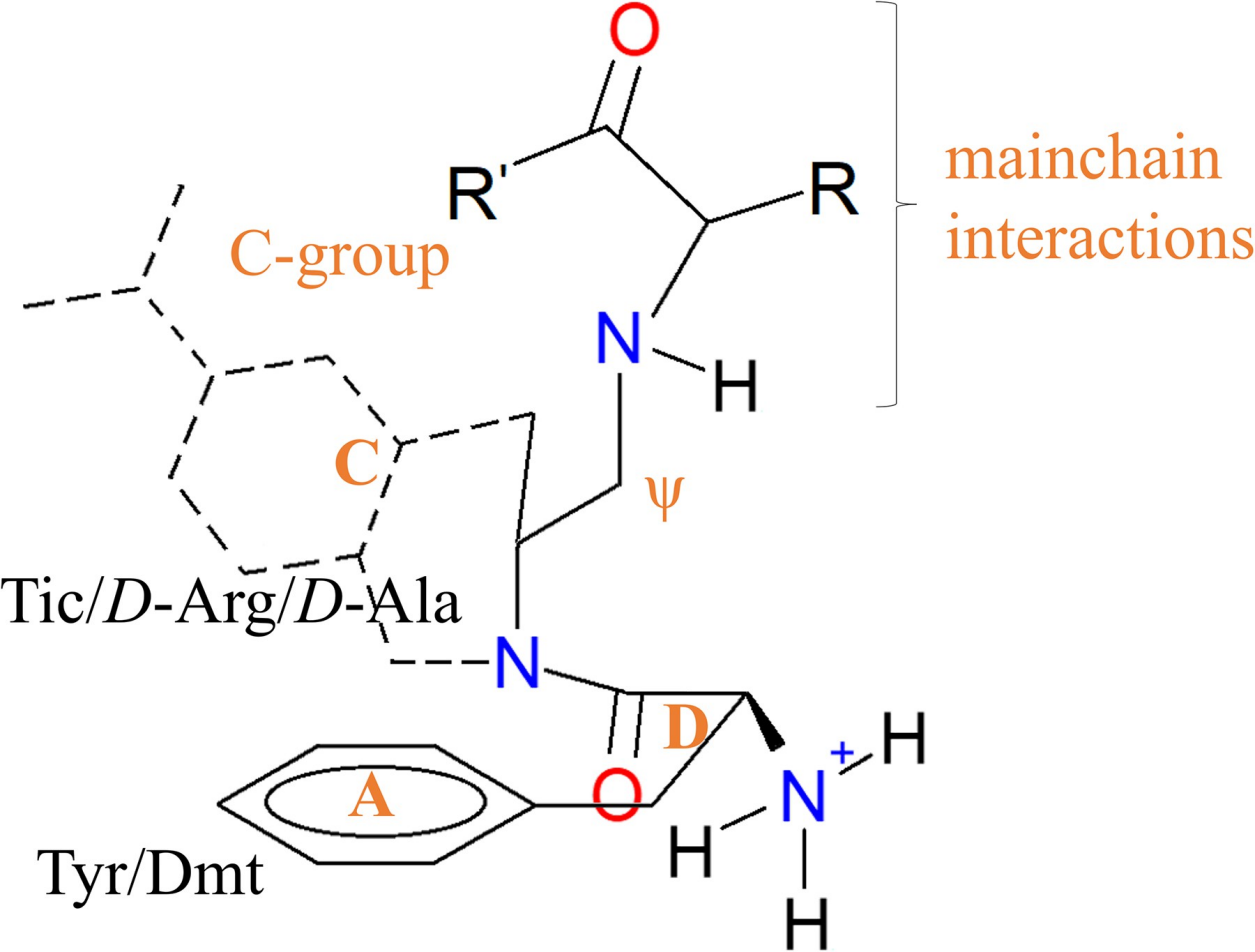
Equivalent scaffold in the peptide ligands, including the pseudopeptide position (ψ) in TIPPψ. Tic: 1,2,3,4-tetrahydroiso-quinoline-2-carboxylic acid, Dmt: 2,6-dimethyltyrosine.

The peptide and non-morphinan ligands have equivalent groups interacting with W284^6.58^. The corresponding bulky groups in the peptides that are equivalent and accomplish the mentioned requirement are *i*) the Tic^2^-Phe^3^ aromatic rings of DIPP-NH_2_ and TIPPψ, *ii*) the *bis*-(trifluoromethyl)benzyl cap in KGCHM07, *iii*) the Phe^3^ of Compound 4 and deltorphin II, and *iii*) the *N*,*N*-diethylbenzamide of DPI287 and BW373U86. In DOR, the residues that interact with those bulky moieties are I277^6.51^, F280^6.54^, V281^6.55^, W284^6.58^, R291^ECL3^, and L300^7.35^ (Fig 9 and S8 and S10–S14 Figs). A notable difference between the selective peptides DIPP-NH_2_ and KGCHM07, and enkephalin L, is the lack of bulky groups as sidechains in positions 2 and 3 in the latter, carrying only the Phe^4^ and Leu^5^/Met^5^ that do not successfully meet the interactions with W2846.58. Several endogenous opioid ligands share the N-terminal sequence (Tyr-Gly-Gly-Phe-) with enkephalins: endorphins contain the enkephalin M sequence, while dynorphins and neoendorphins contain the enkephalin L sequence. These ligands are all non-selective and can bind to δ, μ, and κ receptors with varying affinities, and the interactions established with the C-terminus residues, beyond the Tyr-Gly-Gly-Phe—sequence, might compensate the interactions and let the non-selective binding.

As enkephalins, the smaller morphinan ligands nalorphine, naloxone, and morphine lack any bulky group attached to ring C, and they have instead alcohol, ketone, and/or alkene functions: nalorphine and morphine have 7α-hydroxyl and 8,9-unsaturation, whilst naloxone has solely a 6-ketone (See Fig 8) and they do not interact directly with W284^6.58^. Those ligands are not selective to DOR, and those function variations are mainly in direct contact with Y129^3.33^ in our δ complexes, forming water-mediated interactions that extend to H278^6.52^.

The protonated amino of Phe^3^ in TIPPψ also forms cation-polar contacts with Q105^2.60^ as part of another hydrophobic cluster, along with W114^ECL1^, V124^3.28^, L125^3.29^, and C198^ECL2^. The residue Phe^4^ of DIPP-NH_2_ is closer to I304^7.39^, as buprenorphine does, and Y109^2.64^, whilst Phe^4^ of TIPPψ interacts with R291^ECL3^. The C-terminus of DIPP-NH_2_ forms hydrogen bonds with Q105^2.60^, and that of TIPPψ with R291^ECL3^ and K214^5.39^. The steric hindered DIPP-NH_2_ and TIPPψ ligands interact through the tetrahydroisoquinoline ring of Tic^2^, with K214^5.39^ and W284^6.58^, whereas its carbonyl group and the protonated amino of Phe^3^, respectively, establish a water-bridged and salt bridge with Q105^2.60^ and D128^3.32^ (S11 Fig). The corresponding bulky group in Cmp4 is the sidechain of Phe^5^ and carries an equivalent to the morphine 6α-hydroxyl as the sidechain of Thr^4^ (see S10 Fig). DIPP-NH_2_ does not possess ionic groups at the main chain beyond the N-terminus that forms the D128^3.32^ bridge. Conversely, TIPPψ possesses two additional ionic groups: the protonated amino of the pseudopeptide bond in Phe^3^, and the carboxylate at the C-terminus, which also interacts with D128^3.32^, and the protonated sidechain of K214^5.39^, in similar manner as enkephalin L, whose bulky groups constituted by the sidechains of Phe^4^ and Leu^5^ (that having less steric hindrance due Gly^2^ and Gly^3^ of ENKL), interact lesser with the hydrophobic cluster. KGCHM07 establishes interactions with its amide groups predominantly with Q105^2.60^, and closer contact with D128^3.32^ than DPI287, through the protonated nitrogen along the cMD sampling, but this tendency inverts in GaMD. These findings explain what the identity of the residue positions 4–7 in homolog peptides, determines the ability to interact with DOR. The dynorphines, that contain the N-terminus sequence Tyr-Gly-Gly-Phe-Leu-Arg-Arg- -, have higher affinity to κ rather δ receptor, where the Arg residues contrast with the hydrophobic interactions that we describe for DOR-interacting peptides.

The two full agonist peptides, deltorphin II (DLTR2) and ENKL, exhibited more fluctuation of their Tyr^1^ residue than the other peptides, even KGCHM07, displacing their protonated nitrogen from the hallmark D128^3.32^ interaction. DLTR2 interacts through its peptide bonds and its C-amidated terminus with Q105^2.60^, in a similar manner as DIPP-NH_2_ C-amidated end, while ENKL only with the peptide bonds (S12 Fig). ENKL and the inverse agonist TIPPψ, both with a carboxylate end, do not establish the same interactions. While the enkephalin C-terminus is located frequently in the center of the pore, the pseudopeptide C-terminus interacts with K214^5.39^ in a similar way as the Asp^4^ of DLTR2. Thus, for the peptide ligands, it seems to be relevant to the interactions with Q105^2.60^ and K214^5.39^. Some of the mentioned interactions of the ligands are also depicted in S13 and S14 Figs.

### III. Water molecules penetrate the pore in the full and inverse agonized systems

**Fig 10:**
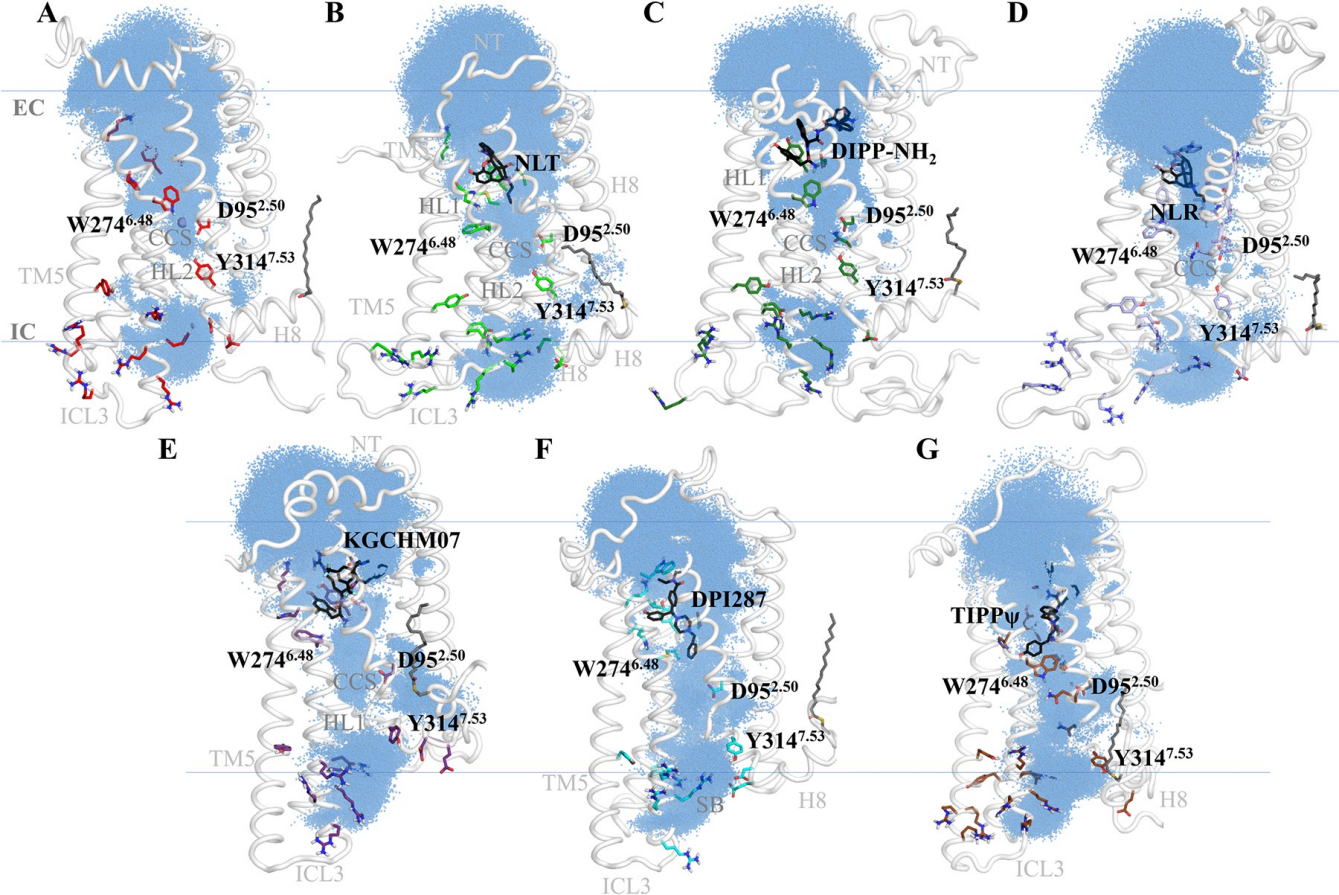
Water dynamics in DOR systems. The cumulative water molecule presence is displayed as blue-colored dots, and the notable regions are highlighted. (A) Apo-δ system, (B) δ-NLT, (C) δ-DIPP-NH_2_, (D) δ-NLR, (E) δ-KGCHM07, (F) δ-DPI287 y (G) δ-TIPPψ. In the apo and TIPPψ systems, the hydrophobic layer 1 (HL1) is hydrated in the surrounding of H278^6.52^ and W274^6.48^, whilst the rest of the systems are dehydrated at this region and under the hydrophobic substructure of the ligands. Only the apo system interacts with a sodium cation at the central coordination site (CCS). At the hydrophobic layer 2 (HL2), the apo, NLT, DIPP-NH_2_, and NLR systems, there is a wide dehydrated region, whilst in KGCHM07, it is slightly hydrated, and in the DPI287 and TIPPψ complexes, the layer is fully hydrated.

Like other Class A GPCRs, the functional state switching of DOR is related to the water presence within the interhelix pore [116, 117] (Fig 10). Our main non-agonized simulated systems (apo, naltrindole, DIPP-NH_2,_ and nalorphine complexes) are characterized by prolonged dehydration of the hydrophobic layer 2 (HL2) at the vicinity of Y318^7.53^, triggered by a distinctive upward rotation of the Y318^7.53^ sidechain. In contrast, in our agonized simulated systems (both active and inactive, DPI287, KGCHM07, and TIPPψ complexes), the HL2 region is hydrated, and the Y318^7.53^ sidechain is oriented outwards the helix bundle (S15 Fig). However, the complex with KGCHM07 maintained a certain degree of HL2 dehydration through the cMD; during the GaMD, the complex thoroughly hydrated HL2. The GaMD simulated systems in complex with DPI287 and TIPPψ consistently maintained the continuous water presence through the pore (S16 Fig).

As part of the DOR conformational changes resulting from pore hydration, in the non-agonized systems, the arginine-rich intracellular region, ends of TM5 and TM6, orient their sidechains towards the pore, facing toward the water molecules. In contrast, in the agonized systems, sidechain orientation in this region is dispersed. Although the interacting Gα_*i*_ protein does not carry a highly negative charge density, the Y318^7.53^ rotamer and the arginine orientations may block the incoming Gα_*i*_ interactions with DOR. Interestingly, a similar water hydration pattern is conserved in our set of additional systems, including the naloxone complexes (data not shown). These findings support the described functional states of the systems, in agreement with the reported features of active receptors.

### IV. The central coordination site (CCS) chelates a sodium ion with a water molecule shell and S135^3.39^ as the first shell

**Fig 11:**
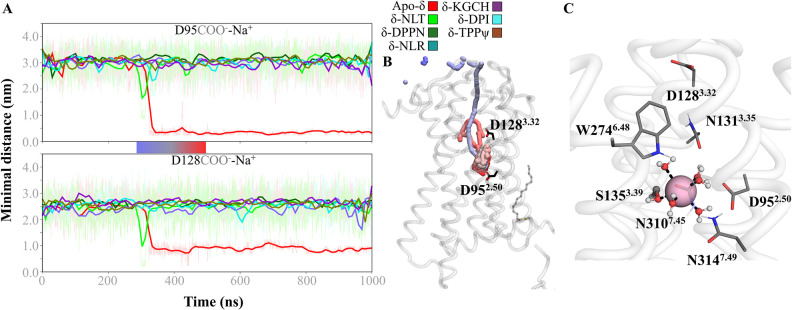
Coordination of Na^+^ in the CCS. A. Minimal distance of any sodium cation to D95^2.50^ and D128^3.32^ residues, where only the apo-δ system chelates Na^+^ in the CCS. B. Trajectory between 300 to 500 ns of cMD, showing the path of the Na^+^ from the EC side to the CCS. C. Representative conformer of the Na^+^ within CSS, where establishes a hexacoordinated geometry: one coordination bond with S135^3.39^ and five bonds with water molecules.

In our main simulated systems, the Central Coordination System (CCS) displays notable changes in its constitution. As it has been found [53, 118, 119], the sodium cation in the CCS functions as an allosteric inactivator of GPCRs. The conserved residue D^2.50^ [106], along with N310^7.45^ and S311^7.41^, plays a pivotal role in the cation coordination, which is a key feature of the Conserved Cationic Site (CCS) in Class A GPCRs. These residues are critical for maintaining the structural integrity of the receptor and are involved in its functional regulation. This particular triad forms a microdomain that is known to interact with sodium ions, which have been shown to function as allosteric modulators influencing receptor activity. Our focus on this triad stems from their established involvement in GPCR activation mechanisms, which has been supported by extensive literature, including seminal work by Ballesteros and Weinstein, as cited. In our apo-δ, a Na^+^ ion interacts within the CCS, entering the pore through the extracellular side (Fig 11), remaining complexed to the last of the long simulation, in the short cMD, and the two subsequent GaMD replicates (S17 Fig). In the two main active systems, δ-DPI287 and δ-KGCHM07, an additional Na^+^ interaction is formed in one short cMD and one GaMD replicate, respectively, with D322^H8^ and E323^H8^, although it is not fully sustained in the time. In our antagonist complexes (NLT and DIPP-NH_2_), the sidechain of S135^3.39^ rotated away from the CCS, diminishing the first shell of coordination observed in the apo-system (S18 Fig). In the agonist-bounded systems, DPI287 and KGCHM07, the sidechain of N314^7.49^ uncouples from the D95^2.50^, N310^7.45^, and S311^7.41^ triad. Interestingly, in the inverse-agonized system with TIPPψ, N310^7.45^ and N314^7.49^ are displaced downward due to the unfolding and movement of TM7, in addition to the S310^7.41^ rotation. The sodium coordination solely in the apo-system is in agreement with the fact that DOR possesses constitutive activity, since the cation is required to diminishing the chance of spontaneous activation of the apo-receptor system.

In summary, we hallmark the differences among our simulated systems as follows:

1. The agonist- and antagonist-bounded systems exhibit differences regarding the peptide and small-molecule nature of the ligand classes. Nevertheless, several features are shared within the agonized and antagonized systems, predominantly around the 0.8 μs of the respective simulations.
2. TM5 extends its helicity through the IC region, TM7 experiences a partial unfolding at the conserved NP^7.50^XXY motif, and H8 changes mostly in folding and/or orientation, in a full and inverse agonist-bounded dependent manner, whilst TM5 and TM6 move outward each other in the apo-system and in presence of antagonist or the partial agonist ligands. At the EC region, TM1 and TM2 increase contacts substantially in the presence of a full agonist and lose them in the antagonist, partial agonist, and inverse agonist complexes.
3. The variety of interactions between the ligands and the receptor is higher when it is a full or inverse agonist and lesser with antagonists. The ligand DPI287 (and its relatives BW373U86 and PN6047), and naloxone (three replicates) establish a predominant contact with N131^3.35^, implicated in the biased activation. DPI287 shares conformational ensemble similarities such as the biased agonist PN6047. A bulky substructure in the ligand, contacting a hydrophobic region at the extracellular ends of TM6 and TM7, is a common feature of the δ-selective ligands. The lack of this feature is found in non-selective ligands, such as nalorphine, morphine, naloxone, and even buprenorphine.
4. The water presence in the interhelix pore is profuse in the agonist complexes, especially with DPI287 during the long cMD sampling. In the antagonized complexes and apo-system, there is a dehydrated region termed hydrophobic layer 2, that is hydrated in the agonized systems. In the GaMD simulations, the agonized systems exhibit fully hydration of that region, and the non-agonized remain the dehydration pattern.
5. Only the apo-system interacts with a Na^+^ ion in the interhelix pore, within a central region where the conserved residue D95^2.50^ is located; the CCS, entering from the EC side. The cation interacts with a water molecule network, N131^3.35^, and adjacent to the orthosteric site and the transmission switch, as relevant features.

With our findings, we contribute to the characterization of the functional state ensembles of the δ receptor, identifying key conformational changes that collectively describe the influence of each type of ligand on it, and the role of their most notable structural features in interacting with the receptor. Future directions in delta receptor study comprise the characterization of the biased mechanisms of activation, and the interactions with transducers. An experimental next step could be the confirmation of our computational findings, such as the distinctive binding pose ensemble of naloxone, the biased-like activity of DPI287, and further studies on the pharmacological importance of the delta receptor in health and disease.

## Conclusions

DOR pharmacology takes advantage due to its innovative modulation of narcotic and antidepressant effects. Further data will become available since its use and misuse as a narcotic lead to severe adverse effects, and nowadays, it has an impact on the current opioid crisis dangerously raises. Out of the μ pharmacology, the δ and κ are also emerging intensively as fields of study. Our study has shed light on the structural pharmacology of the δ-opioid receptor (DOR), which can be used as a foundation for developing more targeted therapeutics. By understanding the interactions at the extracellular tryptophan residue and the characteristics of the amine-anchored substituent, we have laid the groundwork for designing selective ligands that could significantly reduce undesirable μ-opioid receptor (MOR) activation. This level of selectivity is crucial for creating safer analgesics that have a reduced potential for abuse and side effects. This directly addresses one of the major challenges in current pain management and opioid addiction. Specifically, we propose experimental testing of criteria derived from our findings for selectivity and non-activating ligand profiles. This has led to the design of ligands with specific scaffolds, such as morphindole, morphobenzofuran, or morphoquinoline, which are predicted to engage DOR preferentially over MOR due to their structural complementarity with the DOR binding site. Our work has also explored the impact of amine-anchored substituents, particularly those that are electron-dense, on ligand selectivity. By leveraging quantum chemical calculations within our computer-aided design framework, we have identified functional groups that enhance DOR interaction without activating MOR, thereby reducing the risk of undesired side effects. Our δ-receptor systems are consistent with the most relevant reported conformational features, with exceptionally well-defined characteristics at the ensembles, and in conjunction with the characterized ligand activities, let to underline the particular roles and interactions of the compounds bound to DOR. While our current findings offer significant insights into the behavior of the delta-opioid receptor, we recognize the need for further exploration. Future studies will aim to delve into dimer modeling and the intricate dynamics of G protein and β-arrestin complexes. These efforts are anticipated to shed light on the allosteric modulation mechanisms and the multi-faceted nature of receptor signaling. Such advancements are expected to pave the way for the development of novel therapeutic agents targeting the delta-opioid receptor, with the potential for more effective and safer treatments for pain and addiction.

## Supporting information

S1 Fig Structures of all the δ ligands studied in this work. They include morphinan core-containing ligands, 4-benzhydrylpiperazine/4-benzhydrylidenepiperidine, and peptides/pseudopeptides.
(TIF)

S2 Fig RMSD profiles of the main systems. (A) The root mean square deviation (RMSD) of the backbone of the transmembrane domain (TMD) of δ receptor, is plotted against the simulation time. The profile of the active complex with KHCHM07 is changing along time, as expected for an activating system. (B) Comparisons of RMSD distributions of apo-receptor system replicates, showing the overlap between them.
(TIF)

S3 Fig Representative conformers for the simulations of all systems. The conformers were aligned within the initial structure embedded in the membrane. It is evident that in the apo and the antagonized systems (in the top row, red and green colors) the IC end of TM5 and TM6 are farthest each other, and in many cases, are partially unfolded in favor to larger ICL3. In contrast, in all the active systems (the middle row, purple and bluish colors) the IC ends of TM5 and TM6 are closer and extend their helicity in detriment of ICL3. In some cases, TM7 and H8 experience an extensive torsion and unfolding. The partial/biased and inverse agonists (last row) possess mixed conformational features.
(TIF)

S4 Fig Changes in TM1-TM2 and TM6-TM7. (A) Secondary structure of TM7. As seen in the representative conformers, the apo, antagonized and partial systems conserve the folding in TM7 (in the latter, an incipient kink is forming next to the allyl group of nalorphine). In contrast, in the two agonized and inactive systems, TM7 partially unfolds at the NPXXY motif. Matrix distance differences with respect the apo system, (A) between TM1-TM2 and (B) TM6-TM7, with a cutoff of 1.0 nm. The distance-based contacts in the non-active systems are very close that the apo, with lesser contacts near to the EC side in the nalorphine system, and greater within the same region in the TIPPψ complex. In contrast, in the two active systems the contacts between TM1 and TM2 are uppermost. In the naltrindole complex, there are lesser contacts at the IC side between TM6 and TM7, whilst in DIPP-NH_2_ system there are all similar than apo-DOR. In the nalorphine complex, like in DOR-NLT, the contacts are similar in addition to a region of minor interactions near to the EC side. For the KGCHM07, DPI287 and TIPPψ systems, the contacts are greater in the IC side.
(TIF)

S5 Fig Conformational findings among the systems that we studied. The conformations are taken from the clustering analysis of the TMD. Superpositions of (A) the main systems: apo-δ and the complexes with naltrindole, DIPP-NH_2_, nalorphine, DPI287, KGCHM07 and TIPPψ, where notable conformational and distinctive patterns are evident. (B) Superposition of the apo-δ and δ complexes of all the antagonists studied and nalorphine (excepting naloxone replicates). The quite-similarity among the conformers of those systems is notable, as well as the helix extension of the IC end of TM5, very similar to the apo-δ. (C) Antagonist complexes, including the three replicates of naloxone system. The EC end of TM1 varies among the naloxone replicates and nalorphine conformers. (D) Nalorphine, and agonist complexes: KGCHM07, DPI287, BW373U86, morphine, enkephalin L and deltorphin II. Within the several conformational differences, the inclination of the IC end of TM5 and TM6 are the most relevant and related with the change of functional state. (E) Nalorphine, agonist complexes, including the biased agonist PN6047. (F) All the agonized systems, including the two inverse agonist complexes: TIPPψ and SYK657. The representative conformers of the inactive states are similar to the active ones, although the former reach states of dynamical equilibrium, detected from the RMSD profiles.
(TIF)

S6 Fig Ligand interactions of all the cMD simulated systems of DOR. The array shows the standardized frequency of contacts within δ receptor, from almost null interaction (in white color) to a predominant contact (in blue color). The systems are sorted in agreement to the activity of the ligands. i) Y56^1.39^, a residue closer to the orthosteric site, interacts only with the Compound 4 through the naphthylalanine^1^ residue. ii) The conserved D95^2.50^ and S135^3.39^ of the central coordination site only interact with naloxone (NLX), through its protonated amino group, may acting as a sodium cation equivalent, which is known that prevents the receptor activation. iii) The three structurally related ligands, PN6047, DPI287 and BW373U86 share the interaction with A98^2.53^, through the *N*-benzyl or allyl moiety; and only slightly contacting naloxone. iv) The contacts with F104^2.59^, W114^ECL1^ and V124^3.28^ are present with inverse or full agonist peptide ligands (excepting enkephalin L), and it is driven through the Phe^3^ residue of the ligand. The selectivity of the contacting peptides may play a role in that the enkephalin does not interacts with those residues. v) Q105^2.60^ is contacted through the amide only by the peptide ligands, regardless of their activities (TIPPψ, DIPP-NH_2_, Compound 4, KGCHM07, deltorphin II and enkephalin L). vi) D128^3.32^ and M132^3.36^ are contacted by all the studied ligands. vii) Y129^3.33^ interacts predominantly with all but TIPPψ, naloxone, compound 4 and BW373U86. viii) The residue N131^3.35^, implicated in the biased activation through β-arrestin pathway, interacts with naloxone and the related PN6047, DPI287 and BW373U86. ix) K214^5.39^ establishes interaction with both, the ammonium group, and the methylene groups of its sidechain. As saline bridge, it interacts with deltorphin II (with Glu^3^), through water bridge with morphine and KGCHM07 (with *D*-Arg^3^) and with hydrophobic interaction with SYK657, buprenorphine, DIPP-NH_2_ (with Tic^2^), PN6047, DPI287 and BW373U86. x) The transmission switch W274^6.48^ establishes direct contacts with TIPPψ, naloxone, DIPP-NH_2_, DPI287, morphine, KGCHM07, deltorphin II and enkephalin L. These interactions are driven by the residue 1 of the peptide ligands (Tyr^1^ and Dmt^1^), the rings A, B/D and the 17-alkyl group of the morphinans (naloxone and morphine), and the equivalent benzyl group of DPI287. xi) H278^6.52^ predominantly forms water bridges with the phenol function of ligands, and direct hydrogen bonds in a lesser extent. xii) W284^6.60^ interacts with the benzofuran and indole functions in SYK657 and naltrindole respectively, the 7-bulky group of buprenorphine, the disubstituted amide in PN6047, DPI287 and BW373U86, and Phe^3^ and/or Phe^4^, and Phe^5^ of compound 4 of the peptide ligands, excepting the inverse agonist TIPPψ. xiii) L300^7.35^ interacts with all the peptide ligands, although slightly with enkephalin L, and the morphinans but buprenorphine, naloxone, nalorphine and morphine. All of these ligands that do not form a predominant interaction with L300^7.35^ are non-selective to the opioid receptors. xiv) I304 interact with all the ligands excepting naloxone, nalorphine, and slightly with DPI287 and BW373U86. xv) Y3087.43 establishes interactions with all the morphinans, slightly with nalorphine, BW373U86 and the peptide agonists.
(TIF)

S7 Fig Interactions of the morphinan ligands. (A) The agonist morphine (MRP) and the partial agonist nalorphine (NLR), both non-selective, lacking a large, hydrophobic C-group. The two ligands share the interactions excepting the 17-group, that in nalorphine is an allyl function that interacts with G307^7.42^ and W274^6.48^. (B) The antagonists naltrindole (NLT) and buprenorphine (BPNF), only the prior being a selective ligand; and SYK656, that possess a benzofuran fused system than the indole in NLT. The C-group of buprenorphine, hydrophobic and voluminous as the selectivity requirement, is branched from the C2 carbon atom of the 14α-ethane, and it is oriented toward the vestibule of the receptor and Q105^2.60^ and the disulfide bridge, rather than W284^6.58^ within the hydrophobic pocket. The hydroxyl function in the C-group interact slightly with the glutamine and cystine residue. (C) Superposition of all the morphinan ligands, (excepting naloxone), showing resuming the ligand configurations.
(TIF)

S8 Fig Comparison between the partial agonist nalorphine and the antagonist naloxone in the orthosteric site. (A) Superposition of nalorphine and the three replicates of naloxone in the orthosteric site, for the first 400 ns of simulation. (B) The most similar configuration of naloxone complex replicate (termed NLX-1) with respect nalorphine. The allyl group of the former interacts closer with N131^3.35^, whilst that of the latter is closer to M132^3.36^. (C) The replicate NLX-2, where naloxone was translated and rotated towards the central coordination site. (D) The replicate NLX-3, where naloxone only was displaced to the central coordination site. Despite the differences, the overall conformers of NLR and (E) NLX-1 replicate, and (F) NLX-2 and NLX-3 replicates show very similar configurations for the receptor. (H) Comparative pattern of interactions of naloxone replicates, with naltrindole (as functional-similar antagonist) and nalorphine (as structural-like morphinan). The bar plot shows the contacts, that in many cases, and despite the distinctive poses among the ligands, they interact in alike manner. As naloxone binds preferentially to μ-μ dimers [120], and it bears a ketone group rather than hydroxyl, we analyzed replicates and found higher variations in comparison with its related compounds. The unsaturated bond of the *N*^17^-allyl group of naloxone replicate-I establishes a predominant interaction with N131^3.35^ with an average distance 0.288 nm, while in the replicate-II, the 17-group interacts the most with F270^6.44^ at an average distance of 0.284 nm, and in the replicate-III, it tends to extend towards the CCS (in a similar way as the alkyl chain of a ligand within the CB_1_ receptor [113]), interacting mainly with N314^7.48^ (38.9% of the simulation time, at an average distance of 0.274 nm along the last 900 ns), and secondly, with W274^6.48^ (29.9% of the time at 0.276 nm). Rather, the cyclopropyl ring of naltrindole interacts with G307^7.42^, and the alkene of allyl in nalorphine scarcely with G307^7.42^ and W274^6.48^. In the other hand, and unexpectedly, the ring of the *N*^17^-benzyl group in SYK657 establishes hydrophobic interactions with I277^6.51^ and I304^7.39^, whereas the equivalent benzyl function in DPI287 locates closer to M132^3.36^. The configuration of naloxone in the replicate-I is roughly similar to nalorphine one, although the two have many differences in the ensembles. In the three replicates, the rotation of the sidechain of Y129^3.33^ at χ_2_ dihedral is unrestrained, in contrast with the more fixed configuration in the naltrindole and nalorphine systems. While Y129^3.33^ forms a hydrogen bond with the O^6^ and epoxide O of nalorphine, the hydroxyl group of the residue remains far from these atoms in the naloxone systems. The 14-hydroxyl of the replicates interacts with N131^3.35^ during about 200 ns, S135^3.39^ the last 700 ns, and G307^7.42^, respectively, contrasting with that of naltrindole and SYK657, that interact in a high extent with D128^3.32^. Those exclusive patterns of naloxone, with respect the analogues and equivalent groups in our simulations, and knowing its experimental particularities, turns its mechanism as a novel path to antagonize the δ receptor, at least in the monomeric protomer.
(TIF)

S9 Fig Interactions of all agonist ligands in the orthosteric site. (A) The median configurations of the selective agonists DPI287 and BW373U86, and the biased PN6047, of the benzylidenepiperazine class, show a very similar pose. The *N*-attached substituents, namely the 17-groups, share a closer interaction with W274^6.48^ or N131^3.35^ than the peptide counterparts. The contact with the indole ring system of W274^6.48^ is direct for the *N*^17^-(5-thiazolyl) of PN6047 and strictly hydrophobic with the benzyl of DPI287. Due these interactions with the transmission switch that is not shared with BW373U86, we suggest that DPI287 may also a biased agonist. The C-group substructures, the secondary amides that confer their selectivity to DOR, interact with W284^6.58^, in agreement with other selective ligands discussed. Those non-peptide ligands do not interact notably with Q105^2.60^ and the disulfide bridge. (B) Comparison of all the agonist ligands, where is noticeable the agreement of their substructure interactions and configurations, and that the peptide ligands interact with the glutamine and cysteine residues, establishing hydrogen bond and hydrophobic interactions, particularly with the C-amidated end of deltorphin II.
(TIF)

S10 Fig Detailed interactions of the cyclopeptide compound 4 (Cmp4). The residue Tyr^3^ of the ligand, equivalent to ring A, interacts in agreement with the other ligands, and rather the ammonium group, the Trp^2^, Tyr^3^ and Thr^4^ peptide bonds interact with D128^3.32^. Cmp4 establishes several aromatic and hydrophobic interactions beyond the C-group, constituted by the residues Phe^5^ and Thr^4^; forming contacts through Nal^1^ and Trp^2^ with Y56^1.39^, Q106^2.60^ and K108^2.63^, as well as Y308^7.43^, respectively.
(TIF)

S11 Fig Interactions of the non-agonist, peptide ligands. (A) The peptide antagonist ligands DIPP-NH_2_ and Compound 4. (B) DIPP-NH_2_ and the inverse agonist TIPPψ. DIPP-NH_2_ interacts with C198^ECL2^ and Q105^2.60^ through Phe3, while Compound 4 via Nal1, and TIPPψ also through Phe3. The phenol function of ring A is similar in DIPP-NH_2_ and Cmp4, but different in TIPPψ. The cyclic peptide did not interact predominant, and directly with W274^6.48^, whilst TIPPψ did it.
(TIF)

S12 Fig Interactions of the full agonist, peptide ligands. (A) KGCHM07 and enkephalin L (ENKL) in the orthosteric site, from a clustering analysis. The Dmt^1^ residue of KGCHM07 that is equivalent to the morphinan ring A, is placed predominantly at the same configuration, while Tyr^1^ of enkephalin L displaces to W274^6.48^. The N-terminus of the prior interacts with D128^3.32^, whilst that of enkephalin L displaces to TM7. The KGCHM07 residue *D*-Arg^2^ and the C-cap *bis*-(trifluoromethyl)-benzyl (Bz(CF_3_)_2_) function, the C-group, are surrounded by W284^6.58^ and its hydrophobic pocket, in a similar fashion than enkephalin residues Phe^4^ and Leu^5^. Phe^3^ and Gly^3^ constitute a small hydrophobic environment next to Q105^2.60^ and C121^3.25^. (B) KGCHM07 and the selective δ_2_ agonist deltorphin II (DLTRII) in the orthosteric site. The N-terminus of both peptides interact similarly with D128^3.32^. The bulky C-group of deltorphin II is mainly Val^5^, that is located next to Bz(CF_3_)_2_ of KGCHM07 and Leu^5^ residue of the enkephalin. As KGCHM07, deltorphin II interact as hydrophobic via Phe^3^ residue the glutamine and cystine residues, while enkephalin L does it through the mainchain of Gly^3^. As the inverse agonist TIPPψ, the Asp^4^ residue of deltorphin II is positioned toward the protonated, primary ammonium of K214^5.39^. The C-amidated end of DLTRII form hydrogen bond and hydrophobic interactions with Q105^2.60^ and the disulfide bond, whilst ENKL is oriented to the center of the pore vestibule. (C) Superposition of the agonist-peptides KGCHM07, enkephalin L, deltorphin II, and morphine, showing many of the similarities among them within the orthosteric site. (D) Deltorphin II, enkephalin L and the inverse agonist TIPPψ superpositioned for comparison purposes. The divergent configuration of the ligand Tyr1 between the endogenous and inverse agonists are quite similar in the representative configurations. Although both share the C-terminus as carboxylate, each one has it in different orientations; and the interaction with Q105^2.60^ is peptide-type and hydrophobic, respectively.
(TIF)

S13 Fig Detailed interactions of buprenorphine with DOR. The morphinan core establishes interactions as the other members of this class. The bulky substituent, branched from the 18α atom of the ethane bridge, does not interact with W284^6.58^ as the C-group of the selective ligands. This feature may explain the lack of selectivity of buprenorphine to DOR, also binding to MOR. The methoxy function is surrounded by V281^6.55^ and partially by W284^6.58^.
(TIF)

S14 Fig Some ligand interactions with δ receptor. Water-mediated interactions of Y129^3.33^ with the phenol function of (A) naltrindole and (B) KGCHM07. The protonated residues K214^5.39^ and Arg2 are part of the water molecule network. (C) TIPPψ via the phenol function establishes a direct interaction with H278^6.52^, while this residue forms water-mediated bridges with naltrindole as (D) donor and (E) acceptor hydrogen bond, and with (F) DPI287 as hydrogen bond donor and (G) KGCHM07 hydrogen bond donor. (H) The bis-trifluoromethylphenyl function of KGCHM07 is surrounded by the hydrophobic pocket formed by W284^6.58^, V281^6.55^, F280^6.54^, I277^6.51^, and L300^7.35^, that also confers the specificity of the receptor for the bulky 7-substituents.
(TIF)

S15 Fig Dihedral χ_1_ angles for Y318^7.53^. Polar plots for the cMD and GaMD simulations for A and D: Non-agonized systems, B and E: Inverse agonized systems, and C and F: Full agonized systems. The apo, as well as naltrindole, DIPP-NH_2_, nalorphine, naloxone, buprenorphine, and compound 4 complexes, are invariant in their rotamers, oriented towards the CCS. Between the inverse agonists, only the SYK657 complex adopts the non-agonized rotamer, while the TIPPψ complex resembles the full agonized systems, that predominantly possess the outward rotamer. In the cMD sampling, only morphine and enkephalin-L complexes fluctuate in the dihedral angle populations. In the GaMD sampling, one replicate of DPI287 adopts the outward orientation, and the other, the upward rotamer, while the two replicates of KGCHM07 complex adopt the two rotamers.
(TIF)

S16 Fig Water presence in the interhelix pore of the DOR systems. The tendencies of hydration observed in the cMD sampling continue in our accelerated simulations. For (A) apo-δ, (C) δ-NLT, (E) δ-DIPP-NH_2_, and (G) δ-NLR, the water molecules do not penetrate notably into the hydrophobic layer 2 (HL2), where Y318^7.53^ is located. During the GaMD replicates, (B, D, F, and H respectively), HL2 remains with low water molecule interactions. Nevertheless, the main agonized systems, (I) δ-KGCHM07, (K) δ-DPI287 and (M) δ-TIPPψ, that begin the HL2 hydration during our cMD simulations, fully hydrate the interhelix pore during the GaMD sampling (J, L, and N respectively).
(TIF)

S17 Fig Sodium cation interactions in the CCS in apo-δ. The cation is surrounded by a water molecule network that also interact with the transmission switch W274^6.48^ and N131^3.35^.
(TIF)

S18 Fig Central Coordination Site (CCS) in DOR. The residues D95^2.50^, S135^3.39^, N310^7.45^ and N314^7.49^ form the central sodium ion pocket. (A) Median configuration of the CCS. The Apo, naltrindole, DIPP-NH_2_ and nalorphine are similar in the representative configuration, whereas in the TIPPψ the CCS is collapsed due the unfolding and displacement of TM7. (B) The RMSF of the sidechain of those residues (along with C273^6.47^) is lower in the Na^+^-coordinated and antagonized systems, and greater in the agonized systems, with the inverse agonist TIPPψ complex is the highest.
(TIF)

S19 Fig Structure of the ligands considered for the QSAR. **(**A) Morphindole, protonated, (B) morphindole, non-protonated, (C) morphobenzofuran (protonated), (D) morphoquinoline, (E) 7,8-dehydro-4,5-epoxymorphinan, (F) 4,5-epoxymorphinan, (G) 6-(methylene/ oxo)morphinan, and (H) orvinol core. The shift of *N*^17^-methyl to *N*^17^-allyl in the mild-agonist morphine, originates the partial agonist nalorphine; and from the partial agonist oxymorphone to the antagonist naloxone. Also, the shift from *N*^17^-cyclopropylmethyl to *N*^17^-cyclobutylmethyl, from the agonist 6β-naltrexol to the partial agonist nalbuphine [95]; implying this change a voluminous substituent than those of the morphinan antagonists. In agreement with this facts, it was reported [33] than electron-withdrawing groups as *N*^17^-substituents in the DOR antagonists are important for its activity. The shifting of the cyclopropylmethyl group in naltrindole with phenylacetyl produces the full agonist SYK754, and its replacement with benzylsulfonyl and phenethylsulfonyl substituents, the corresponding partial agonists are produced (47.1% and 88.1% of efficacy, respectively, with respect the reference agonist DPDPE). Secondly, the 17-bencenesulfonyl derivative possesses antagonistic activity, while the mesylyl (CH_3_SO_2_-), triflyl (CF_3_SO_2_-), cyclopropylsulfonyl, and vinylsulfonyl substitutions generate partial inverse agonists (-48.8%, -36.1%, -80.5% and -80.2% respectively). And finally, the cyclopropylcarbonyl (SYK623) derivative is a near-full inverse agonist (up to -69% of efficacy with respect SNC80 [121]) Regarding the differences between carboxamides and sulfonamides, it is reported [122] that the former are chemically harder and with lesser dipole moment than the latter. Also, the phenylsulfonyl moiety increases the basicity in both heteroatoms, higher in the N atom and lesser in the O atoms, whereas the trifluoromethanosulfonyl group decreases the basicity over the same atoms, both with respect to the methanosulfonyl substituent. It is more suitable that the N atom in sulfonamides interact with an acidic proton than the carboxamides, therefore interacting stronger with the adjacent 14-hydroxyl group. Those evidence address that: *i*) the decrease in the basicity of N^17^ influences the shift from agonist to inverse agonist, *ii*) the lack of a continuous electron delocalization influences the activating-like activity. In the case of the phenylacetyl and benzylsulfonyl ligands, it seems that the 14-hydroxyl interaction with the sulfonamide N^17^ diminishes the hydrogen bond donor-character to D128^3.32^, and thus decreasing the activator-like activity with respect to the carboxamide, that may interact through the O atom of the amide, which is farther. This effect of the H-bond donor of the 14-hydroxyl may explain the difference between hydrocodone and oxycodone (both with *O*^3^-methylated phenol function), with 39 and 21% of efficacy, respectively [123] (with respect SNC80; also *O*^3^-methylated), but not with hydromorphone and oxymorphone (29 and 33%). The lack of resonance in the 17-groups joined to the 14-hydroxy group that preserves the interaction with the conserved D128^3.32^, seem to be a cause of the activity on DOR at this level. The most electron delocalization may to determine the most non-activating effect, such is seen with the bencenesulfonyl group in comparison with the former agonists. Varying the cyclopropylmethyl with ethyl and propyl in the indole-replaced quinoline analogues (32.4%, 41.2% and 62.4% of efficacy with respect DPDPE [115]) not influences markedly the partial agonist activity. Apparently, the acyclic alkyl substituents or with less torsional energy, favor an agonistic activity. It may be the case of nalbuphine, a δ-partial agonist that carries a 17-cyclobutylmethyl group. The presence of one to three fluorine atoms in the position 2 of the ethyl group within the naltrindole core, displayed partial inverse agonistic effects (-38.1%, -48.6% and -44.8%, with respect DPDPE), whereas those substitutions in the indole-replaced quinoline core, acquire partial agonist activity (27.1% for the monofluorinated, with respect DPDPE) or neutral antagonist profiles [115]. Since the fluorine atoms possess distinctive electronic properties than alkyl groups, *i*.*e*., higher electronegativity and chemical hardness, they are able to attract more electron density affecting directly the C2 ethyl atom. It may create a more-localized electron environment than the alkane partners, and a relative electron density deficiency at the N^17^, impacting the functional activity. In some of the mentioned cases above, the contributions of the groups seem to be roughly additive. Thus, we summarize that an extended electronic delocalization, and/or the diminishing of allyl electrophile-like carbon atoms in the *N*^17^-substituent, is a contribution to an agonist-like activity at this substructure group. The saturation of the morphinan ring C increases slightly the affinity and activation to the opioid receptors with respect to their desaturated derivatives, as is seen in the shift from morphine to dihydromorphine [1] (with 103% to 106% of efficacy) [124]. The placement of the function 6-alcohol to 6-ketone increases the affinity respective of the parent compound, as is evidenced in the shift from dihydromorphine to hydromorphone [1], but decreases its efficacy (73 and 29% with respect SNC80), although preserving both compounds the agonist activity. During the biotransformation reactions, the diastereomeric variations of the transformations from 6-ketones to 6-alcohol and vice-versa, have a profile impact. It have been reported that the 6α-hydroxyl is more efficacious than the 6β-hydroxyl configuration [125] and thus, 6β-naltrexol has decreased efficacy with respect nalbuphine (besides the N^17^ substituent), as we mentioned above. Further variation in the groups attached to the ring C plays a role in the selectivity and functional activity. While ligands with large groups at position 7α are more selective to δ, the smaller and at 7β tend to be non-selective, such as seen in the typical opiate alkaloids. Herein, two features seem to influence the activity: *i*) the hydrogen bond-donor/acceptor property of the group attached to ring C, and *ii*) the electron donor/acceptor character. The ligands that interact with the hydrophobic cluster possess at least, a conjugated or electron-dense group, and sometimes a hydrogen bond donor/acceptor atom attached directly to ring C. When the hydrogen bond-donor is in the bulky C-group, such as the buprenorphine (with 19% of efficacy with respect SNC80 [110]) and its relatives ‒namely nororvinols‒ diprenorphine (a δ-full agonist with a 2-hydroxy-2-propyl substituent and 98.5% of efficacy [124]), etorphine (a δ superagonist with 2-hydroxy-2-pentyl substituent, and 107% of efficacy [124]), RX6007M (a dihydroetorphine analogue with 2-hydroxy-2-pentyl substituent), thienorphine [126], and buprenorphine itself (non-selective δ-antagonists with 2-(2-thienyl)ethyl and 2-hydroxy-3,3-dimethylpentyl groups, respectively) that in course, possess a hydroxyl group within a saturated substituent bounded to position 7β in the ring C; the ring C constitute itself by an ethane or ethene bridge as in the 14α position. As it has been proposed [127], the steric hindrance of a bulky moiety in the position 7, contacting Y109^2.64^ (and negligibly K214^5.39^), establishes a hydrogen bond with buprenorphine in our simulated complex; and the 6α-methoxyl slightly contacting F280^6.54^, V281^6.55^, W284^6.58^, or I304^7.38^. We argue that one source of interference in the activation-related changes underlies in part, by the steric hindrance to establish a hydrogen bond with the C-group, besides a bulky and hydrophobic group, and even more unfavored with 7β substituents. This hydrophobic cluster, along with the vicinity of Q105^2.60^, seems to be an activation-switch. In agreement with this non-selectivity statement, the DPI287-structural related ligands, PN6047 (with *N*, *N*-dimethylcarboxamide as C-group), nor-RWJ394674 [128] (with *N*-ethylcarboxamide group) and DPI3290 (with *N*-(3-methylphenyl), *N*-methylcarboxamide) [129], both bind to DOR and MOR [130], whereas RWJ394674 (with *N*, *N*-diethylcarboxamide) binds preferentially to DOR. Additionally, since morphine has a predominant interaction with I277^6.53^ through its alkene function in ring C, it corresponds approximately with the ethene bridge of the agonist etorphine, and the unsaturated positions 7 and 8 of other morphinan agonists.
(TIF)

S1 Table General description of our additional cMD simulated δ systems. **a.** We conducted three replicates of the naloxone complex to assess a representative conformational ensemble.
(DOCX)

S2 Table QSAR study conducted on the morphinan ligands. Structure of the ligands considered for the QSAR. Structure of (A) morphindoles: naltrindole and derived Hirayama ligand series (light-yellow shaded), (B) sulfonylmorphindoles of Iwamatsu series (light-green shaded), (C) morphobenzofurans: naltriben and its analogue SYK656, (D) morphoquinoline Nemoto series (light-blue shaded), (E) 4,5-epoxymorphinans: morphine, codeine, nalorphine and dehydronalbuphine, (F) 7,8-dihydro-4,5-epoxymorphinans, (G) morphinan-6-one relatives, and (H) dihydronororvinoles.
(DOCX)

S3 Table QSAR Data of the δ-ligands, including the experimental efficacy, the calculated Gibbs energy (G), and the ground state energy (E_0_), as we found relevant for our QSAR study. (DOCX)

S4 Table QSAR Summary of the coefficients of the bivariate polynomial relation of efficacy with Gibbs and basal energy. Note: *** stands for high significance, ** for mild significance, and * for low significance.
(DOCX)

S5 Table QSAR Summary of the polynomial regression of the bivariate polynomial relation of efficacy with Gibbs and basal energy. (DOCX)

S1 File (ZIP)
