# Supplementary figures and images for: Descriptive molecular pharmacology of the δ opioid receptor (DOR): A computational study with structural approach
Source: PLoS One. 2024 Jul 11;19(7):e0304068. doi: 10.1371/journal.pone.0304068 (PMC11239112; doi:10.1371/journal.pone.0304068)

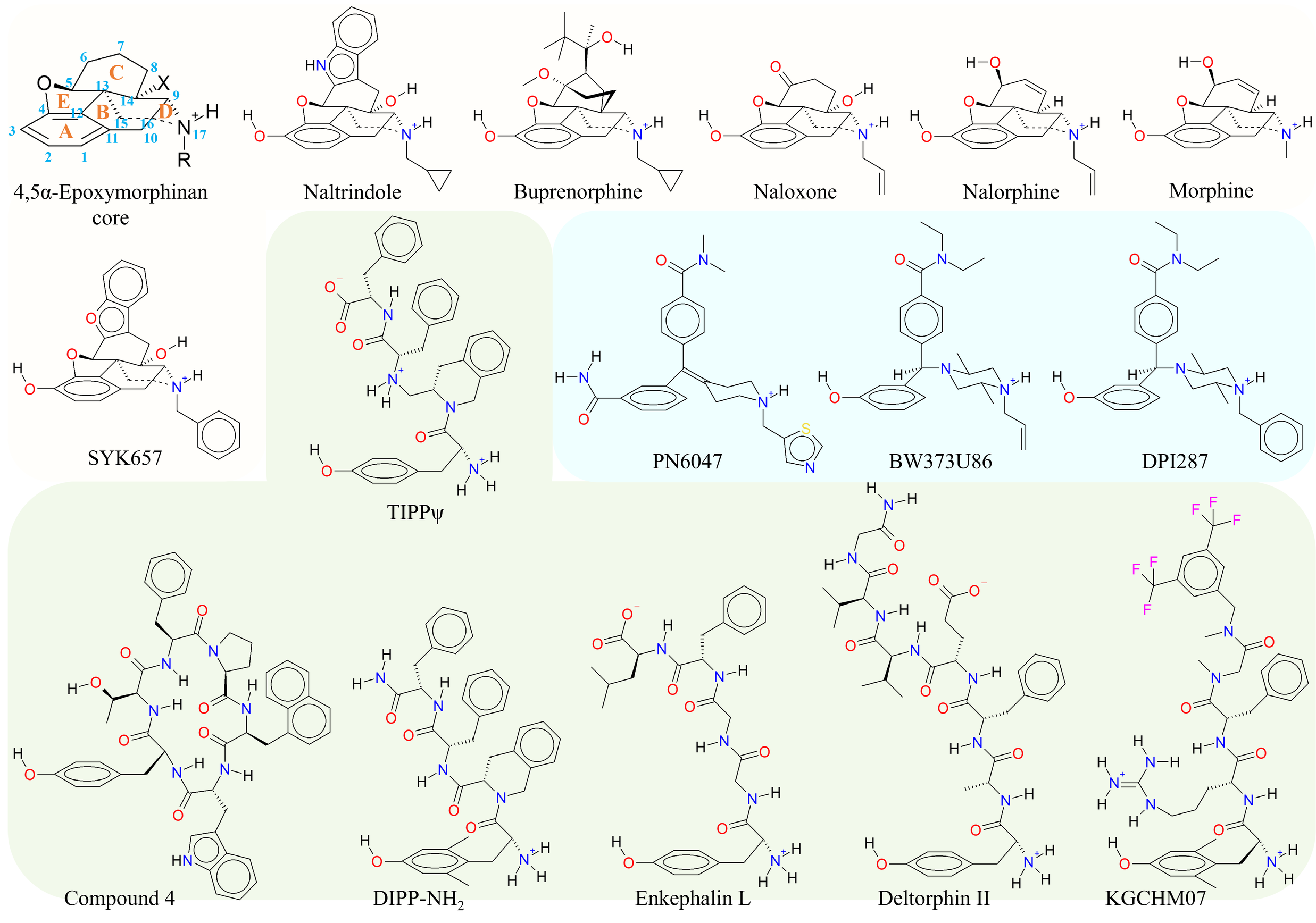

Supplement: S1 Fig — They include morphinan core-containing ligands, 4-benzhydrylpiperazine/4-benzhydrylidenepiperidine, and peptides/pseudopeptides. (TIF) [file pone.0304068.s001.tif]

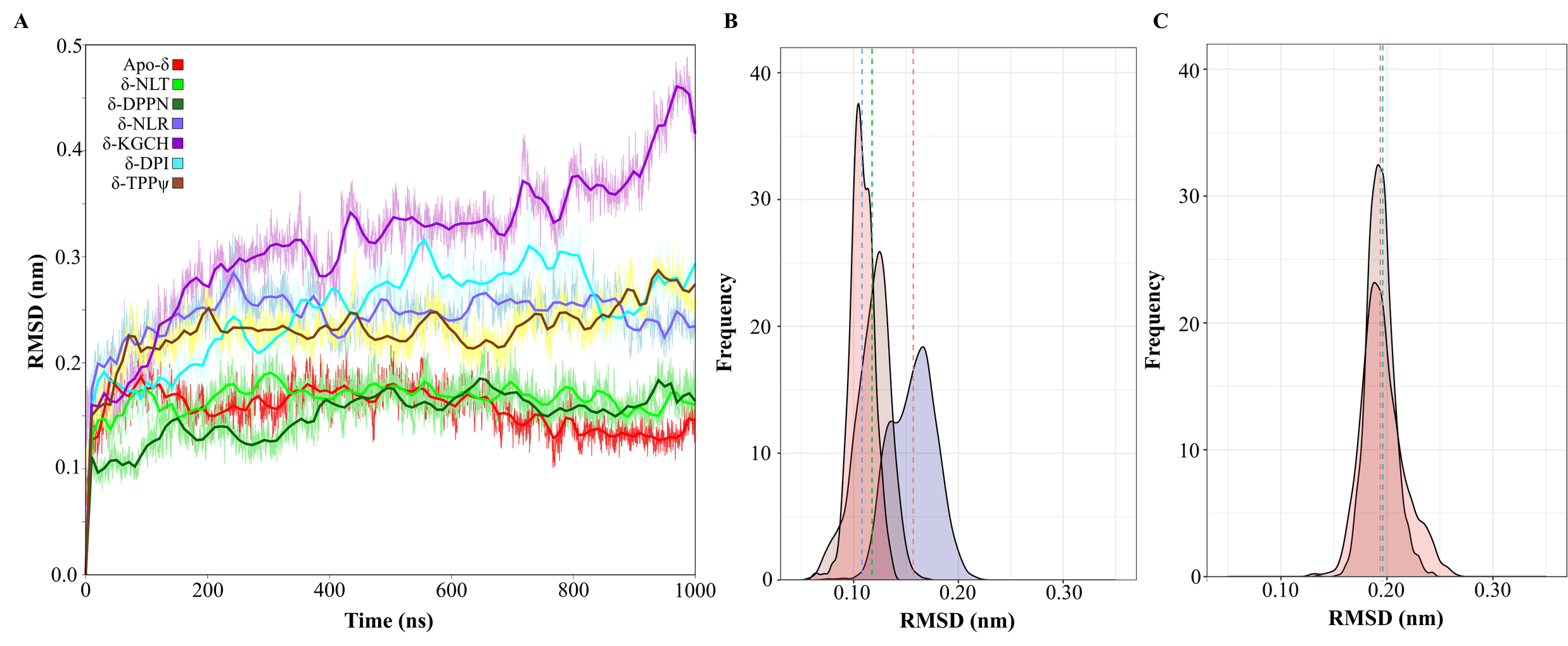

Supplement: S2 Fig — (A) The root mean square deviation (RMSD) of the backbone of the transmembrane domain (TMD) of δ receptor, is plotted against the simulation time. The profile of the active complex with KHCHM07 is changing along time, as expected for an activating system. (B) Comparisons of RMSD distributions of apo-receptor system replicates, showing the overlap between them. (TIF) [file pone.0304068.s002.tif]

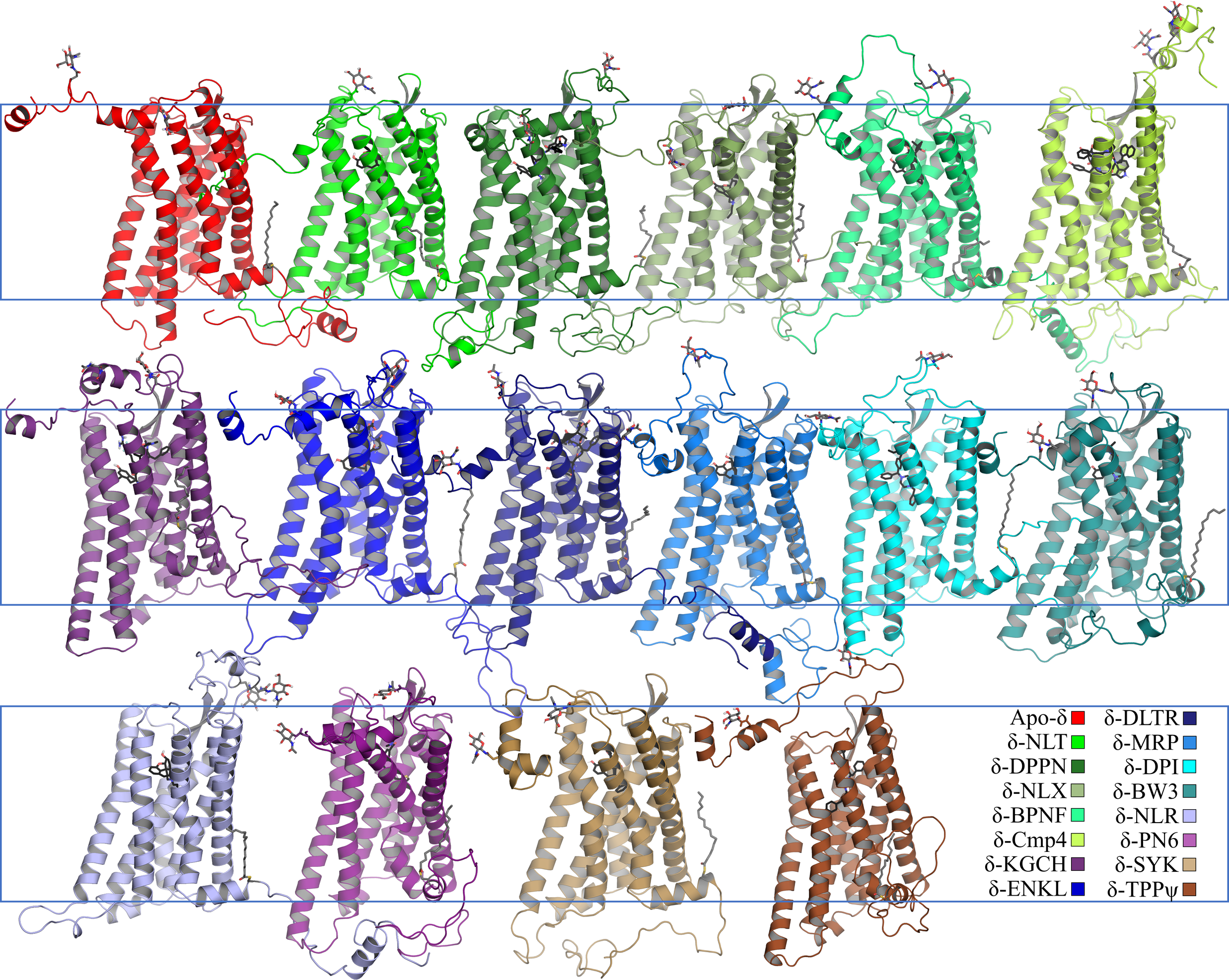

Supplement: S3 Fig — The conformers were aligned within the initial structure embedded in the membrane. It is evident that in the apo and the antagonized systems (in the top row, red and green colors) the IC end of TM5 and TM6 are farthest each other, and in many cases, are partially unfolded in favor to larger ICL3. In contrast, in all the active systems (the middle row, purple and bluish colors) the IC ends of TM5 and TM6 are closer and extend their helicity in detriment of ICL3. In some cases, TM7 and H8 experience an extensive torsion and unfolding. The partial/biased and inverse agonists (last row) possess mixed conformational features. (TIF) [file pone.0304068.s003.tif]

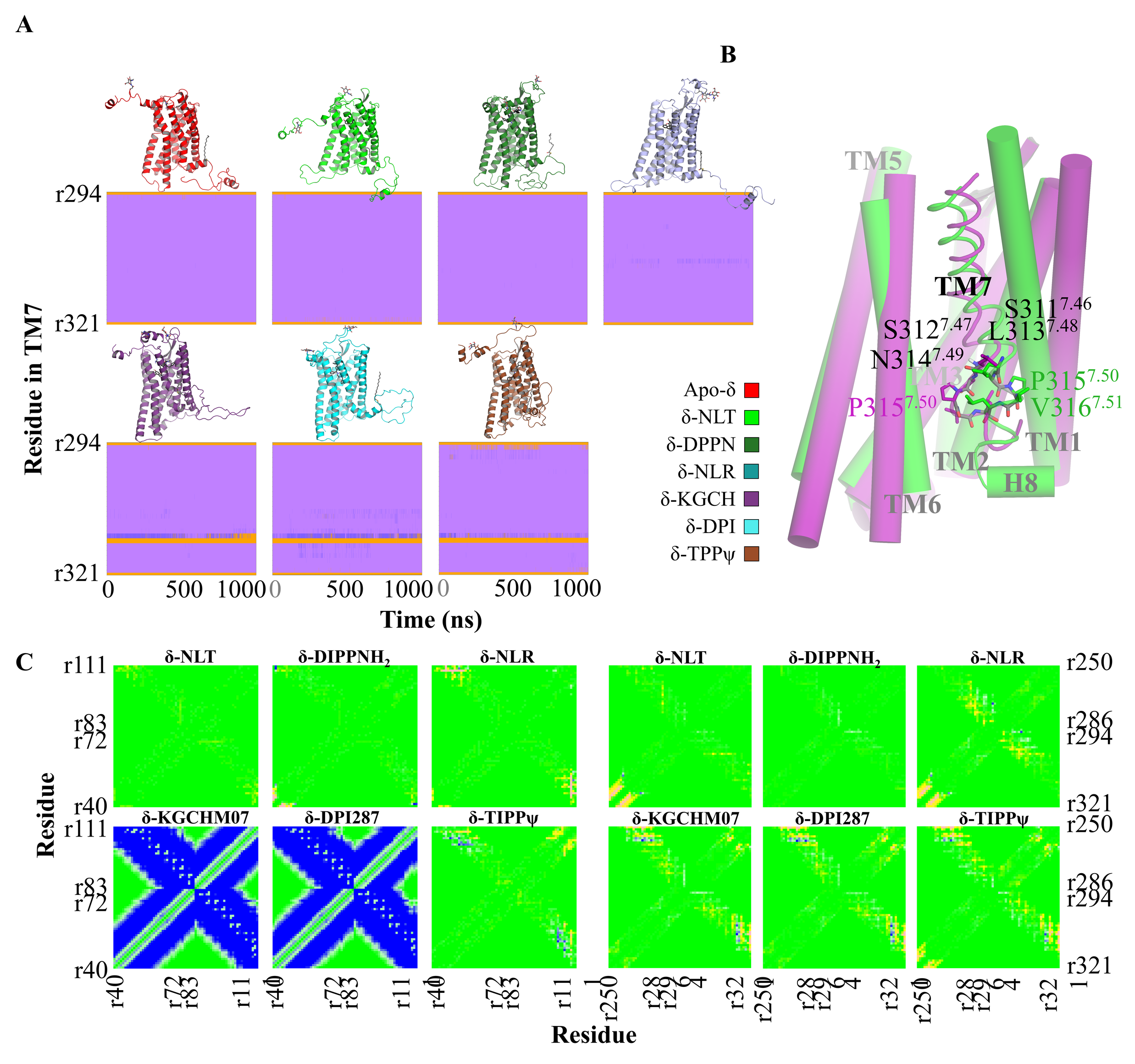

Supplement: S4 Fig — (A) Secondary structure of TM7. As seen in the representative conformers, the apo, antagonized and partial systems conserve the folding in TM7 (in the latter, an incipient kink is forming next to the allyl group of nalorphine). In contrast, in the two agonized and inactive systems, TM7 partially unfolds at the NPXXY motif. Matrix distance differences with respect the apo system, (A) between TM1-TM2 and (B) TM6-TM7, with a cutoff of 1.0 nm. The distance-based contacts in the non-active systems are very close that the apo, with lesser contacts near to the EC side in the nalorphine system, and greater within the same region in the TIPPψ complex. In contrast, in the two active systems the contacts between TM1 and TM2 are uppermost. In the naltrindole complex, there are lesser contacts at the IC side between TM6 and TM7, whilst in DIPP-NH2 system there are all similar than apo-DOR. In the nalorphine complex, like in DOR-NLT, the contacts are similar in addition to a region of minor interactions near to the EC side. For the KGCHM07, DPI287 and TIPPψ systems, the contacts are greater in the IC side. (TIF) [file pone.0304068.s004.tif]

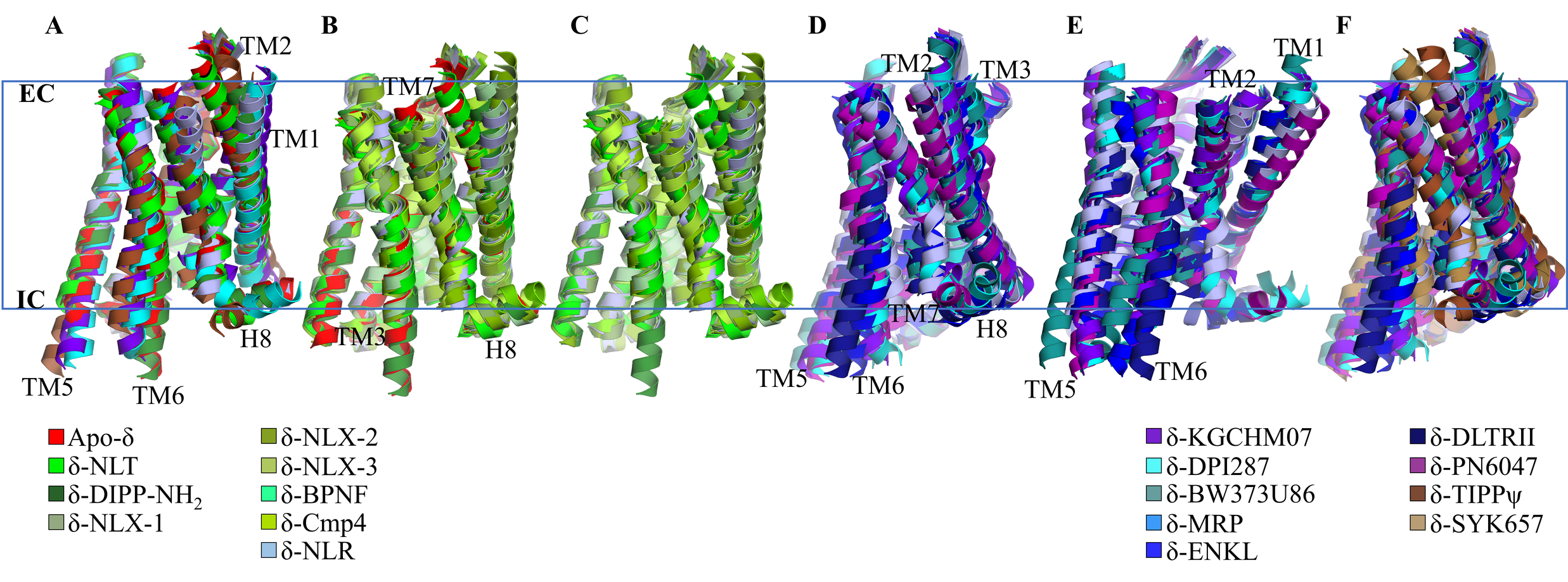

Supplement: S5 Fig — The conformations are taken from the clustering analysis of the TMD. Superpositions of (A) the main systems: apo-δ and the complexes with naltrindole, DIPP-NH2, nalorphine, DPI287, KGCHM07 and TIPPψ, where notable conformational and distinctive patterns are evident. (B) Superposition of the apo-δ and δ complexes of all the antagonists studied and nalorphine (excepting naloxone replicates). The quite-similarity among the conformers of those systems is notable, as well as the helix extension of the IC end of TM5, very similar to the apo-δ. (C) Antagonist complexes, including the three replicates of naloxone system. The EC end of TM1 varies among the naloxone replicates and nalorphine conformers. (D) Nalorphine, and agonist complexes: KGCHM07, DPI287, BW373U86, morphine, enkephalin L and deltorphin II. Within the several conformational differences, the inclination of the IC end of TM5 and TM6 are the most relevant and related with the change of functional state. (E) Nalorphine, agonist complexes, including the biased agonist PN6047. (F) All the agonized systems, including the two inverse agonist complexes: TIPPψ and SYK657. The representative conformers of the inactive states are similar to the active ones, although the former reach states of dynamical equilibrium, detected from the RMSD profiles. (TIF) [file pone.0304068.s005.tif]

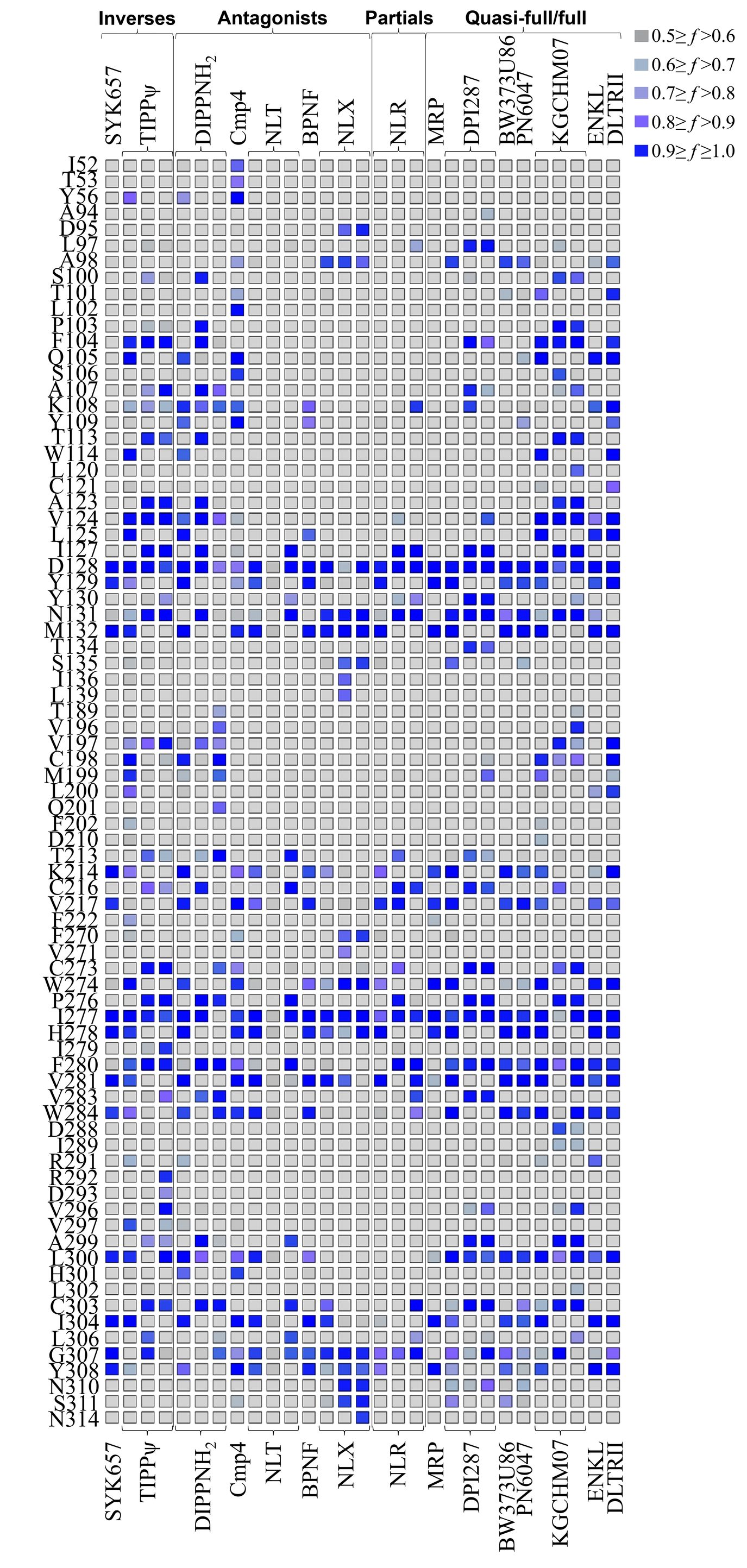

Supplement: S6 Fig — The array shows the standardized frequency of contacts within δ receptor, from almost null interaction (in white color) to a predominant contact (in blue color). The systems are sorted in agreement to the activity of the ligands. i) Y561.39, a residue closer to the orthosteric site, interacts only with the Compound 4 through the naphthylalanine1 residue. ii) The conserved D952.50 and S1353.39 of the central coordination site only interact with naloxone (NLX), through its protonated amino group, may acting as a sodium cation equivalent, which is known that prevents the receptor activation. iii) The three structurally related ligands, PN6047, DPI287 and BW373U86 share the interaction with A982.53, through the N-benzyl or allyl moiety; and only slightly contacting naloxone. iv) The contacts with F1042.59, W114ECL1 and V1243.28 are present with inverse or full agonist peptide ligands (excepting enkephalin L), and it is driven through the Phe3 residue of the ligand. The selectivity of the contacting peptides may play a role in that the enkephalin does not interacts with those residues. v) Q1052.60 is contacted through the amide only by the peptide ligands, regardless of their activities (TIPPψ, DIPP-NH2, Compound 4, KGCHM07, deltorphin II and enkephalin L). vi) D1283.32 and M1323.36 are contacted by all the studied ligands. vii) Y1293.33 interacts predominantly with all but TIPPψ, naloxone, compound 4 and BW373U86. viii) The residue N1313.35, implicated in the biased activation through β-arrestin pathway, interacts with naloxone and the related PN6047, DPI287 and BW373U86. ix) K2145.39 establishes interaction with both, the ammonium group, and the methylene groups of its sidechain. As saline bridge, it interacts with deltorphin II (with Glu3), through water bridge with morphine and KGCHM07 (with D-Arg3) and with hydrophobic interaction with SYK657, buprenorphine, DIPP-NH2 (with Tic2), PN6047, DPI287 and BW373U86. x) The transmission switch W2746.48 establishes direct con [file pone.0304068.s006.tif]

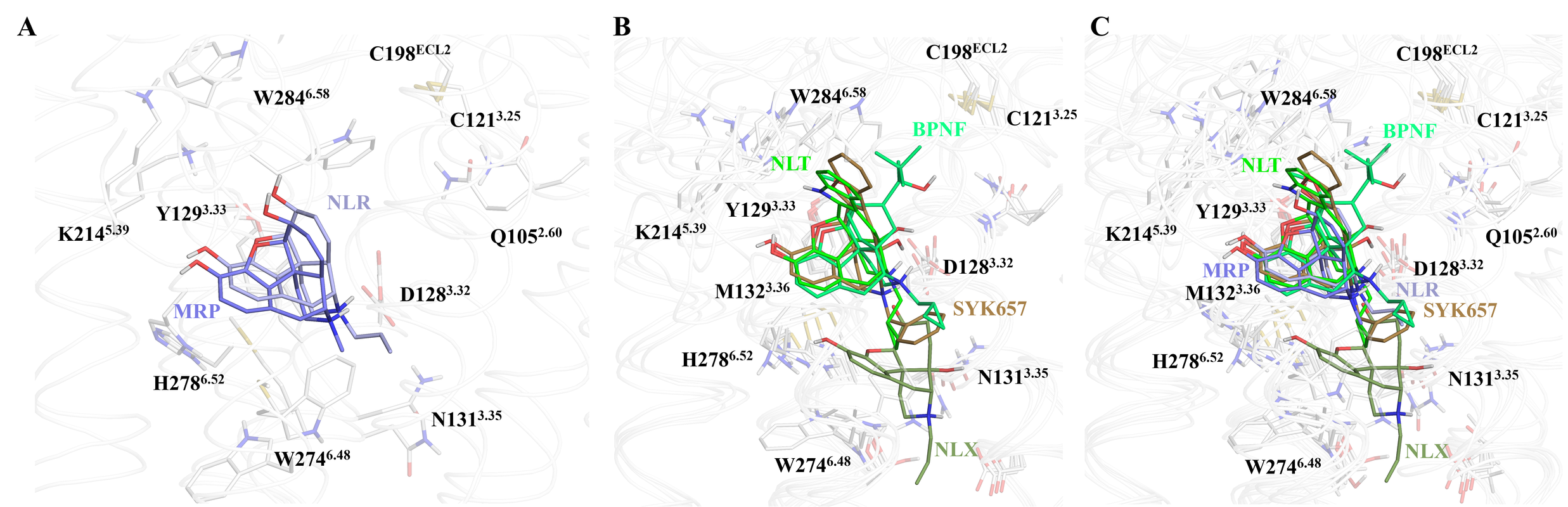

Supplement: S7 Fig — (A) The agonist morphine (MRP) and the partial agonist nalorphine (NLR), both non-selective, lacking a large, hydrophobic C-group. The two ligands share the interactions excepting the 17-group, that in nalorphine is an allyl function that interacts with G3077.42 and W2746.48. (B) The antagonists naltrindole (NLT) and buprenorphine (BPNF), only the prior being a selective ligand; and SYK656, that possess a benzofuran fused system than the indole in NLT. The C-group of buprenorphine, hydrophobic and voluminous as the selectivity requirement, is branched from the C2 carbon atom of the 14α-ethane, and it is oriented toward the vestibule of the receptor and Q1052.60 and the disulfide bridge, rather than W2846.58 within the hydrophobic pocket. The hydroxyl function in the C-group interact slightly with the glutamine and cystine residue. (C) Superposition of all the morphinan ligands, (excepting naloxone), showing resuming the ligand configurations. (TIF) [file pone.0304068.s007.tif]

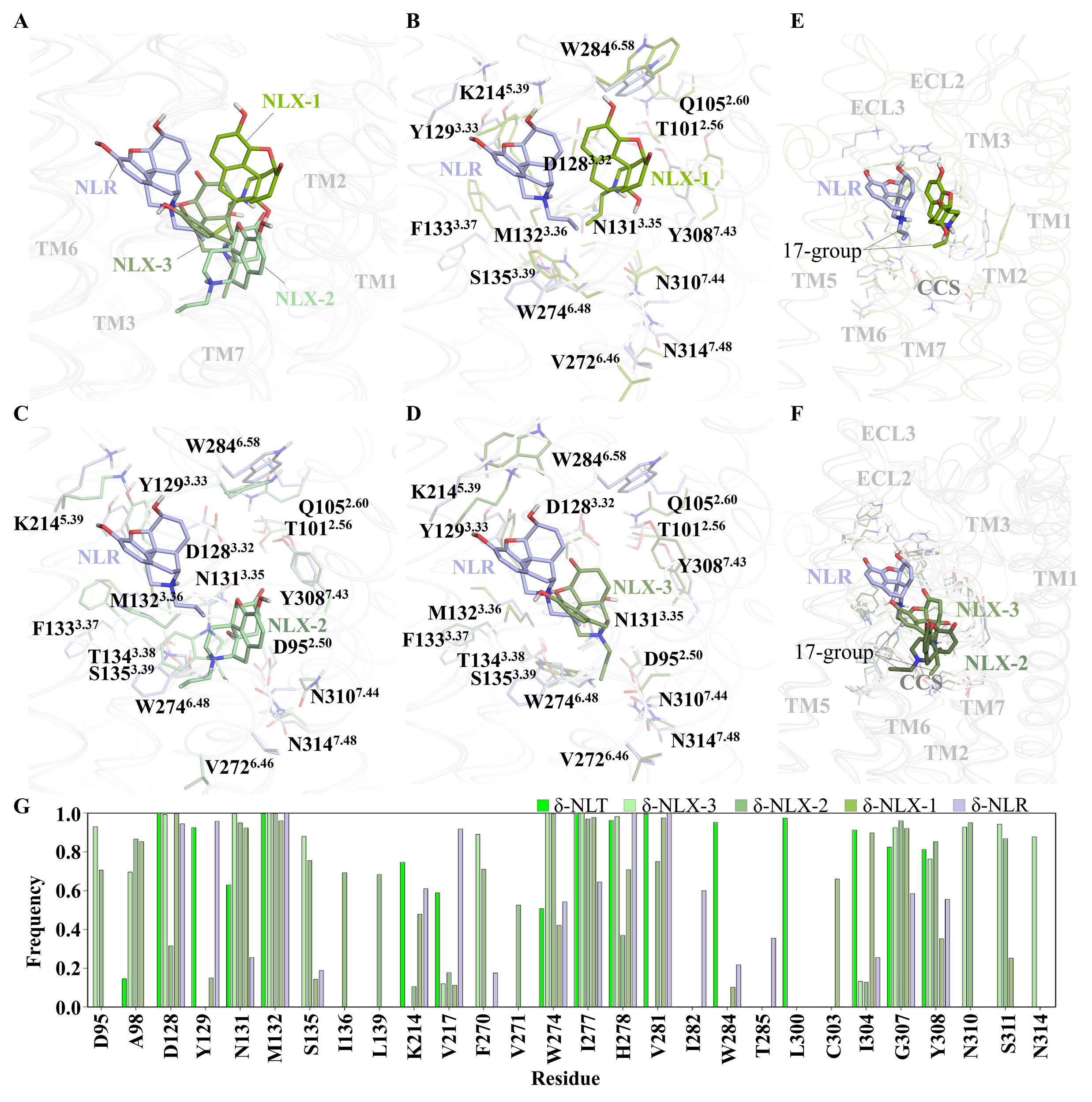

Supplement: S8 Fig — (A) Superposition of nalorphine and the three replicates of naloxone in the orthosteric site, for the first 400 ns of simulation. (B) The most similar configuration of naloxone complex replicate (termed NLX-1) with respect nalorphine. The allyl group of the former interacts closer with N1313.35, whilst that of the latter is closer to M1323.36. (C) The replicate NLX-2, where naloxone was translated and rotated towards the central coordination site. (D) The replicate NLX-3, where naloxone only was displaced to the central coordination site. Despite the differences, the overall conformers of NLR and (E) NLX-1 replicate, and (F) NLX-2 and NLX-3 replicates show very similar configurations for the receptor. (H) Comparative pattern of interactions of naloxone replicates, with naltrindole (as functional-similar antagonist) and nalorphine (as structural-like morphinan). The bar plot shows the contacts, that in many cases, and despite the distinctive poses among the ligands, they interact in alike manner. As naloxone binds preferentially to μ-μ dimers [120], and it bears a ketone group rather than hydroxyl, we analyzed replicates and found higher variations in comparison with its related compounds. The unsaturated bond of the N17-allyl group of naloxone replicate-I establishes a predominant interaction with N1313.35 with an average distance 0.288 nm, while in the replicate-II, the 17-group interacts the most with F2706.44 at an average distance of 0.284 nm, and in the replicate-III, it tends to extend towards the CCS (in a similar way as the alkyl chain of a ligand within the CB1 receptor [113]), interacting mainly with N3147.48 (38.9% of the simulation time, at an average distance of 0.274 nm along the last 900 ns), and secondly, with W2746.48 (29.9% of the time at 0.276 nm). Rather, the cyclopropyl ring of naltrindole interacts with G3077.42, and the alkene of allyl in nalorphine scarcely with G3077.42 and W2746.48. In the other hand, and unexpectedly, the ring of the N17-b [file pone.0304068.s008.tif]

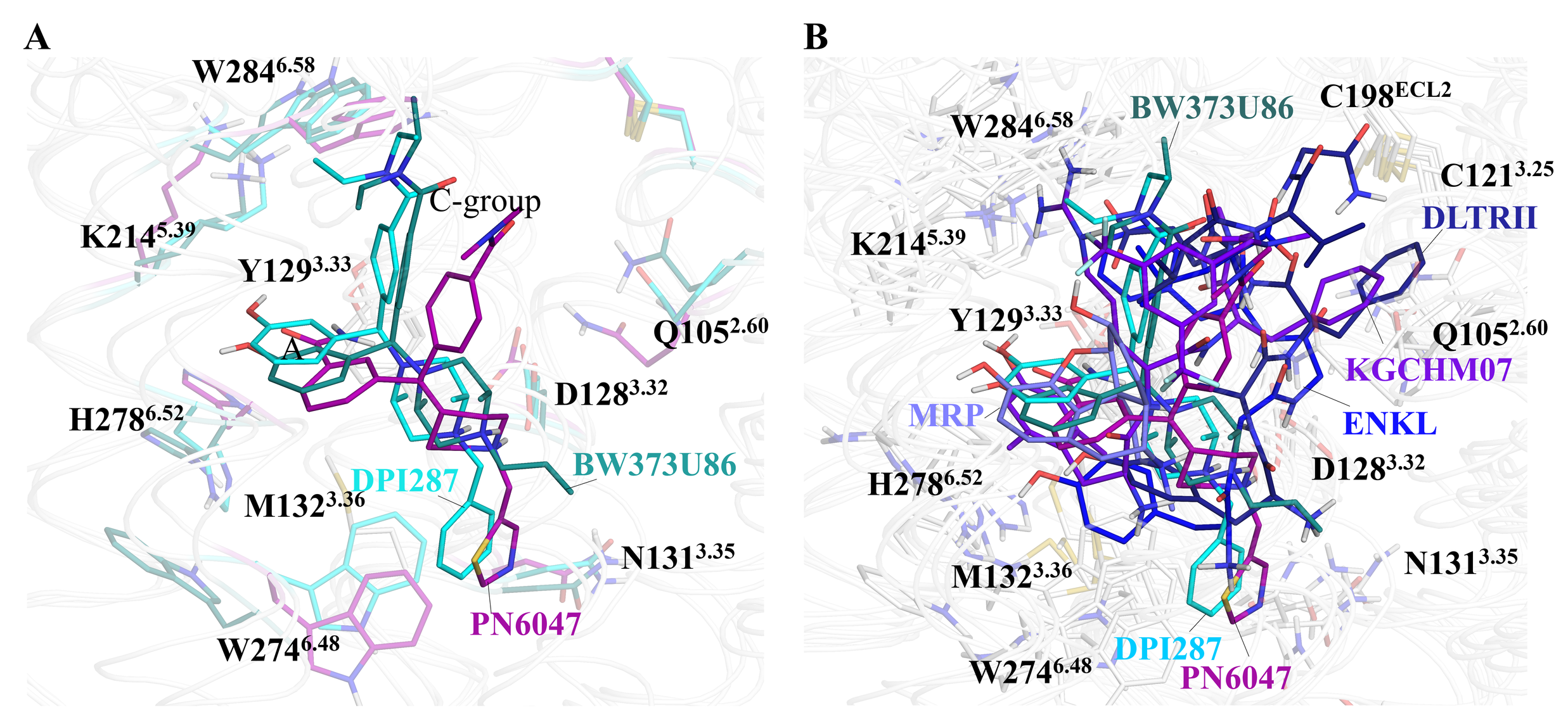

Supplement: S9 Fig — (A) The median configurations of the selective agonists DPI287 and BW373U86, and the biased PN6047, of the benzylidenepiperazine class, show a very similar pose. The N-attached substituents, namely the 17-groups, share a closer interaction with W2746.48 or N1313.35 than the peptide counterparts. The contact with the indole ring system of W2746.48 is direct for the N17-(5-thiazolyl) of PN6047 and strictly hydrophobic with the benzyl of DPI287. Due these interactions with the transmission switch that is not shared with BW373U86, we suggest that DPI287 may also a biased agonist. The C-group substructures, the secondary amides that confer their selectivity to DOR, interact with W2846.58, in agreement with other selective ligands discussed. Those non-peptide ligands do not interact notably with Q1052.60 and the disulfide bridge. (B) Comparison of all the agonist ligands, where is noticeable the agreement of their substructure interactions and configurations, and that the peptide ligands interact with the glutamine and cysteine residues, establishing hydrogen bond and hydrophobic interactions, particularly with the C-amidated end of deltorphin II. (TIF) [file pone.0304068.s009.tif]

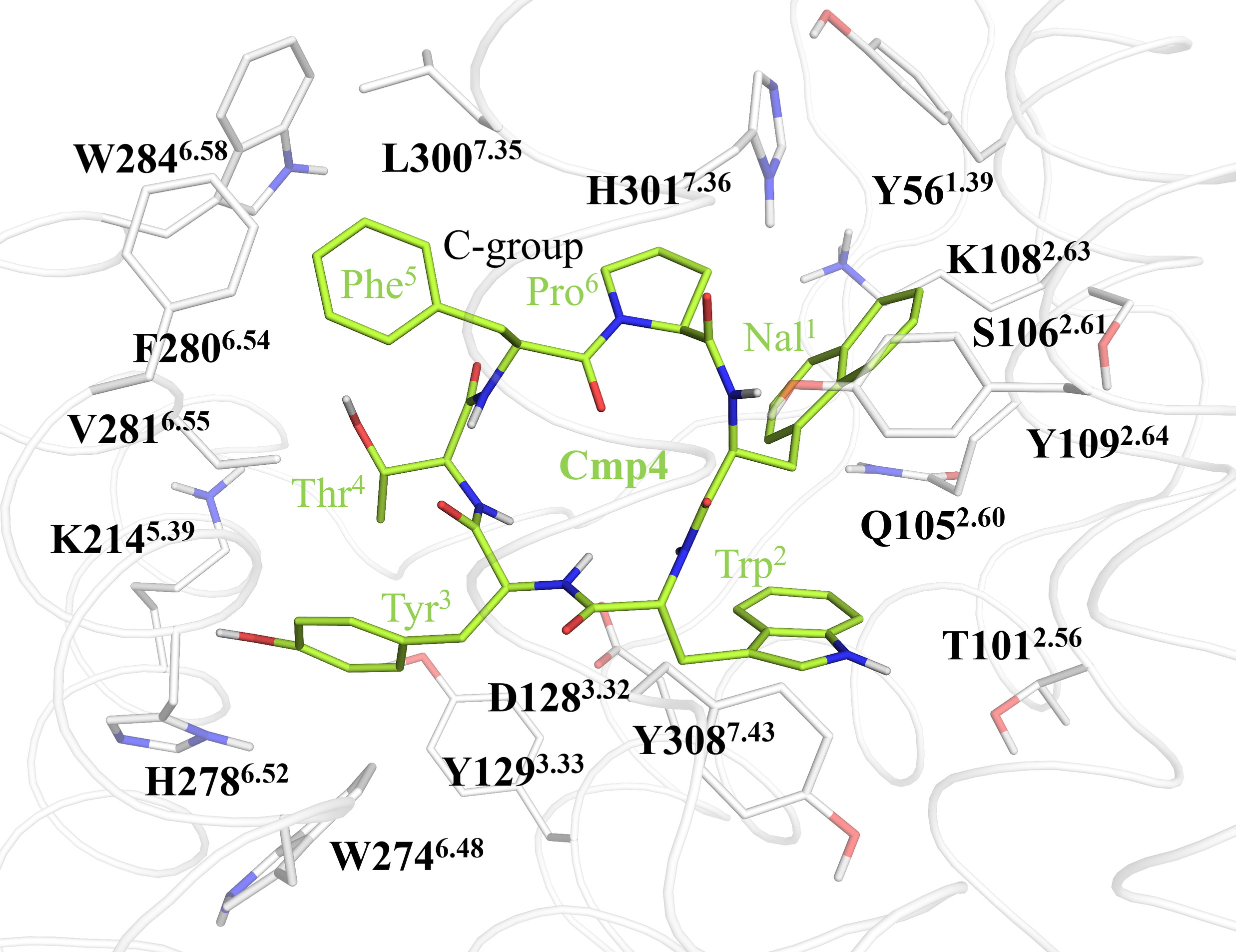

Supplement: S10 Fig — The residue Tyr3 of the ligand, equivalent to ring A, interacts in agreement with the other ligands, and rather the ammonium group, the Trp2, Tyr3 and Thr4 peptide bonds interact with D1283.32. Cmp4 establishes several aromatic and hydrophobic interactions beyond the C-group, constituted by the residues Phe5 and Thr4; forming contacts through Nal1 and Trp2 with Y561.39, Q1062.60 and K1082.63, as well as Y3087.43, respectively. (TIF) [file pone.0304068.s010.tif]

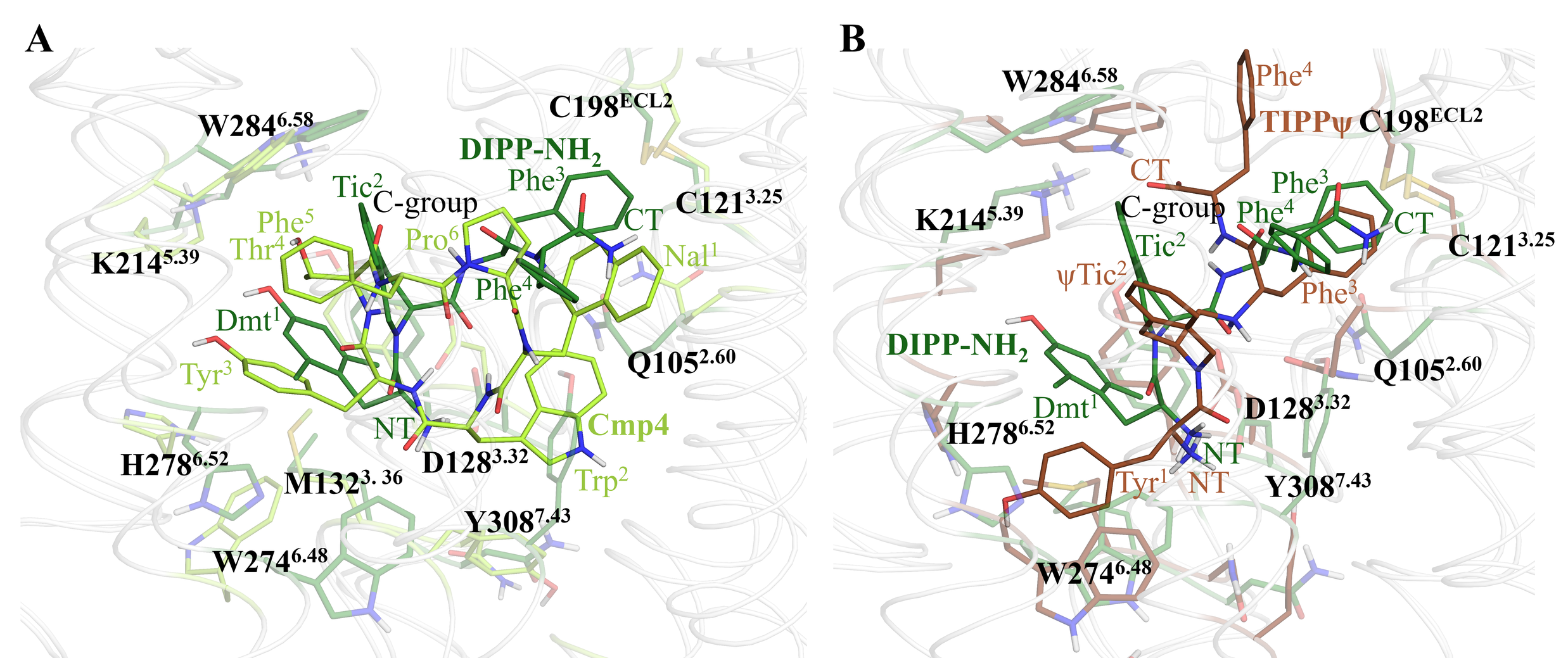

Supplement: S11 Fig — (A) The peptide antagonist ligands DIPP-NH2 and Compound 4. (B) DIPP-NH2 and the inverse agonist TIPPψ. DIPP-NH2 interacts with C198ECL2 and Q1052.60 through Phe3, while Compound 4 via Nal1, and TIPPψ also through Phe3. The phenol function of ring A is similar in DIPP-NH2 and Cmp4, but different in TIPPψ. The cyclic peptide did not interact predominant, and directly with W2746.48, whilst TIPPψ did it. (TIF) [file pone.0304068.s011.tif]

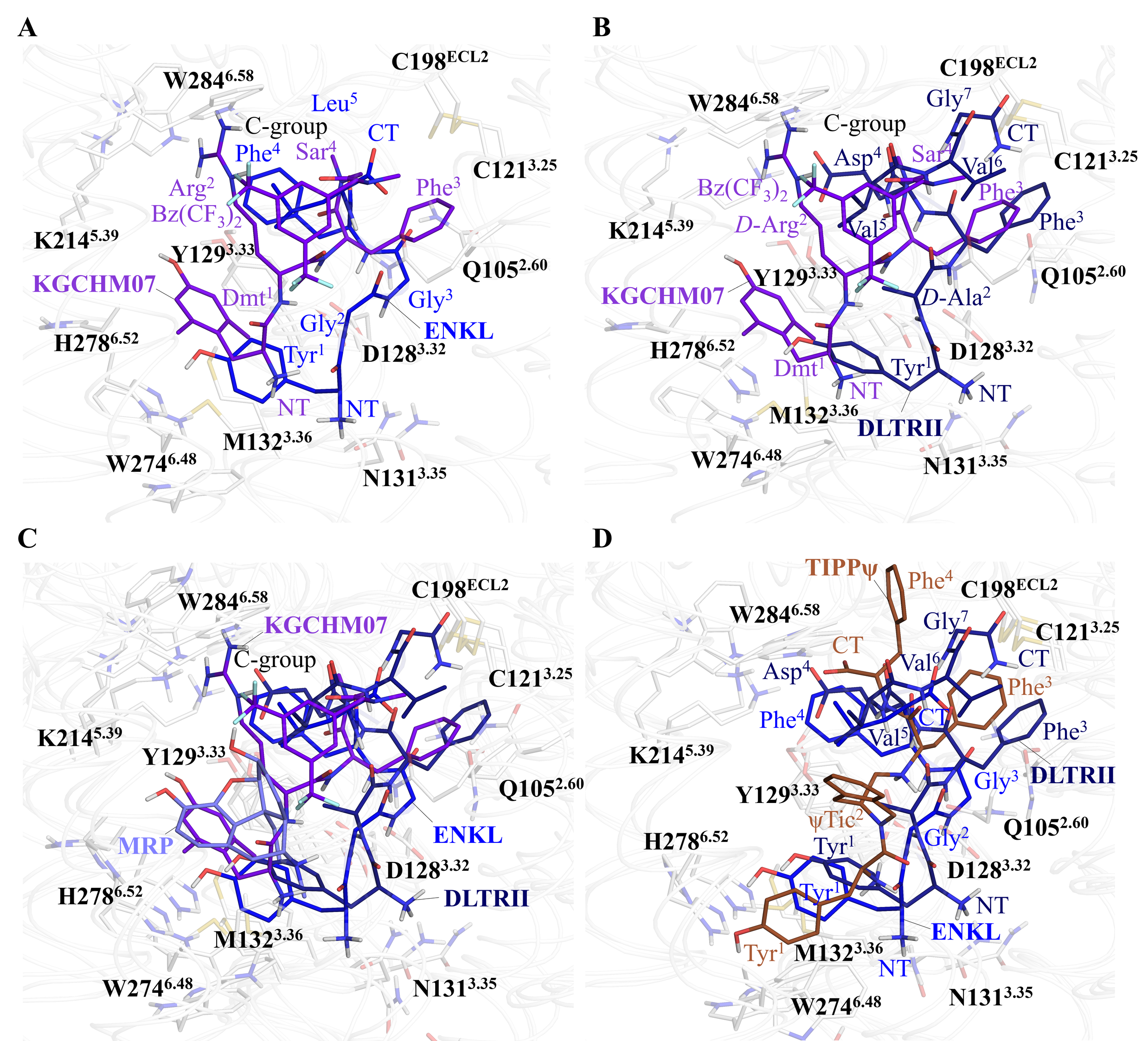

Supplement: S12 Fig — (A) KGCHM07 and enkephalin L (ENKL) in the orthosteric site, from a clustering analysis. The Dmt1 residue of KGCHM07 that is equivalent to the morphinan ring A, is placed predominantly at the same configuration, while Tyr1 of enkephalin L displaces to W2746.48. The N-terminus of the prior interacts with D1283.32, whilst that of enkephalin L displaces to TM7. The KGCHM07 residue D-Arg2 and the C-cap bis-(trifluoromethyl)-benzyl (Bz(CF3)2) function, the C-group, are surrounded by W2846.58 and its hydrophobic pocket, in a similar fashion than enkephalin residues Phe4 and Leu5. Phe3 and Gly3 constitute a small hydrophobic environment next to Q1052.60 and C1213.25. (B) KGCHM07 and the selective δ2 agonist deltorphin II (DLTRII) in the orthosteric site. The N-terminus of both peptides interact similarly with D1283.32. The bulky C-group of deltorphin II is mainly Val5, that is located next to Bz(CF3)2 of KGCHM07 and Leu5 residue of the enkephalin. As KGCHM07, deltorphin II interact as hydrophobic via Phe3 residue the glutamine and cystine residues, while enkephalin L does it through the mainchain of Gly3. As the inverse agonist TIPPψ, the Asp4 residue of deltorphin II is positioned toward the protonated, primary ammonium of K2145.39. The C-amidated end of DLTRII form hydrogen bond and hydrophobic interactions with Q1052.60 and the disulfide bond, whilst ENKL is oriented to the center of the pore vestibule. (C) Superposition of the agonist-peptides KGCHM07, enkephalin L, deltorphin II, and morphine, showing many of the similarities among them within the orthosteric site. (D) Deltorphin II, enkephalin L and the inverse agonist TIPPψ superpositioned for comparison purposes. The divergent configuration of the ligand Tyr1 between the endogenous and inverse agonists are quite similar in the representative configurations. Although both share the C-terminus as carboxylate, each one has it in different orientations; and the interaction with Q1052.60 is peptide-type and hydrophobic, [file pone.0304068.s012.tif]

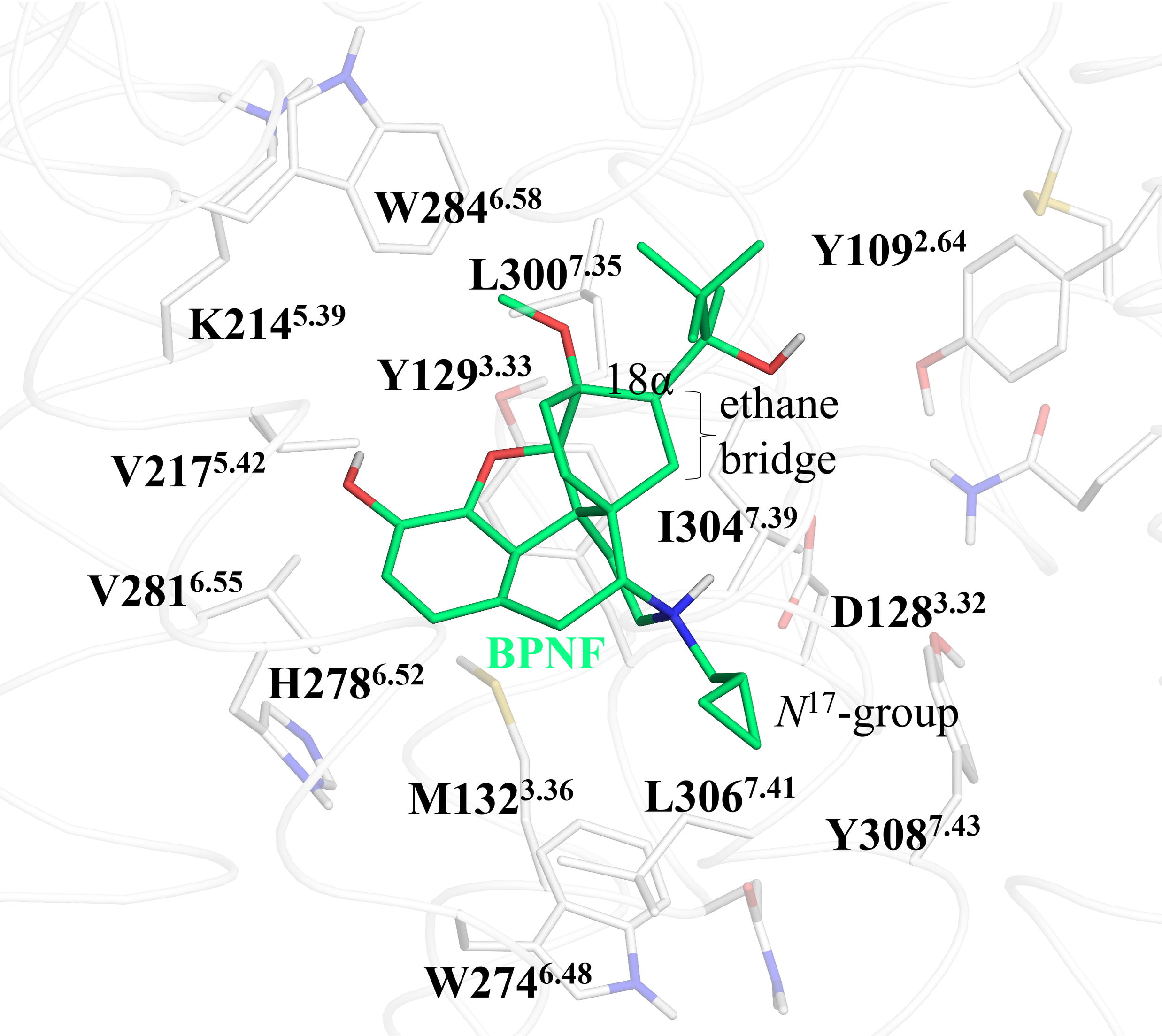

Supplement: S13 Fig — The morphinan core establishes interactions as the other members of this class. The bulky substituent, branched from the 18α atom of the ethane bridge, does not interact with W2846.58 as the C-group of the selective ligands. This feature may explain the lack of selectivity of buprenorphine to DOR, also binding to MOR. The methoxy function is surrounded by V2816.55 and partially by W2846.58. (TIF) [file pone.0304068.s013.tif]

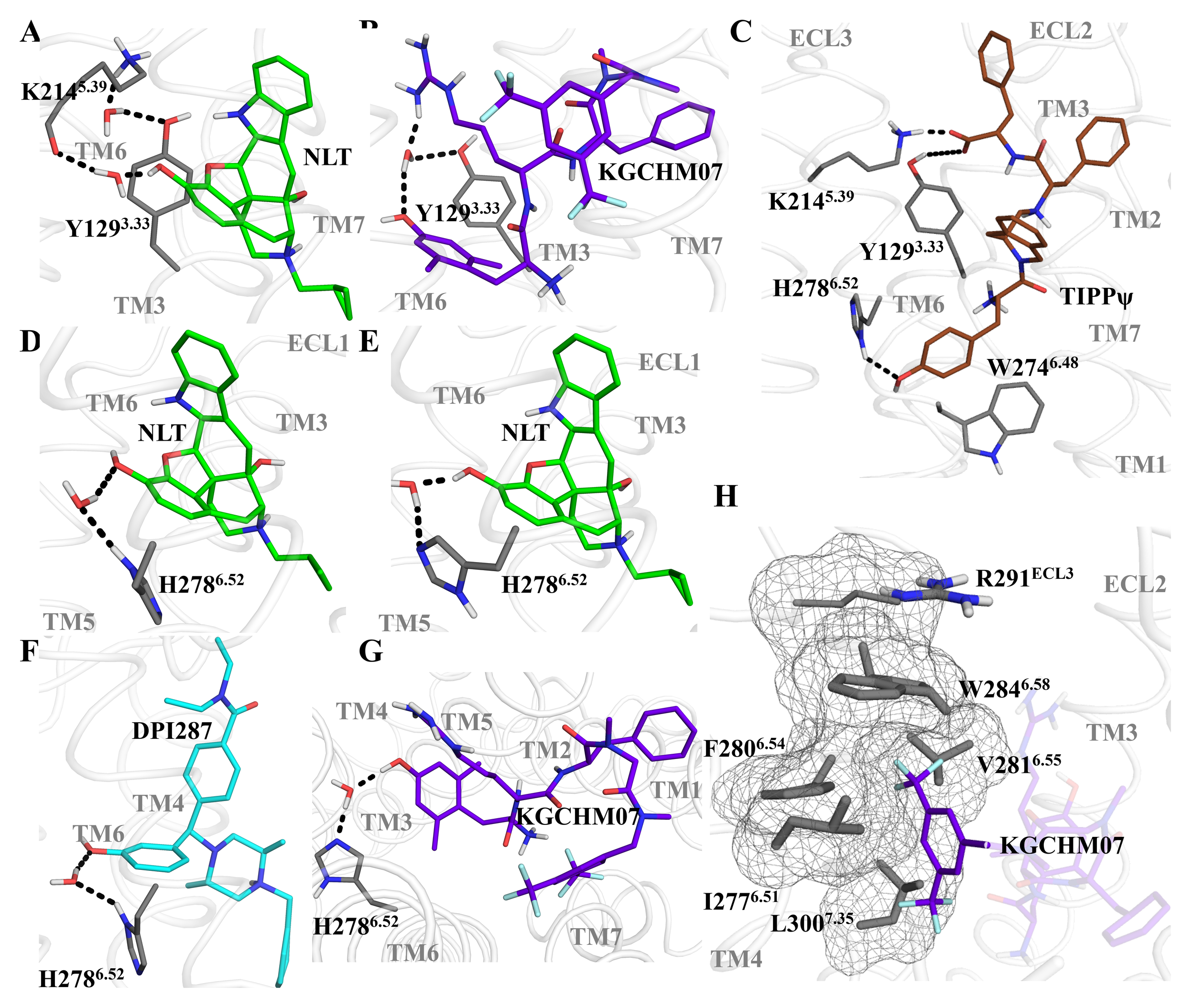

Supplement: S14 Fig — Water-mediated interactions of Y1293.33 with the phenol function of (A) naltrindole and (B) KGCHM07. The protonated residues K2145.39 and Arg2 are part of the water molecule network. (C) TIPPψ via the phenol function establishes a direct interaction with H2786.52, while this residue forms water-mediated bridges with naltrindole as (D) donor and (E) acceptor hydrogen bond, and with (F) DPI287 as hydrogen bond donor and (G) KGCHM07 hydrogen bond donor. (H) The bis-trifluoromethylphenyl function of KGCHM07 is surrounded by the hydrophobic pocket formed by W2846.58, V2816.55, F2806.54, I2776.51, and L3007.35, that also confers the specificity of the receptor for the bulky 7-substituents. (TIF) [file pone.0304068.s014.tif]

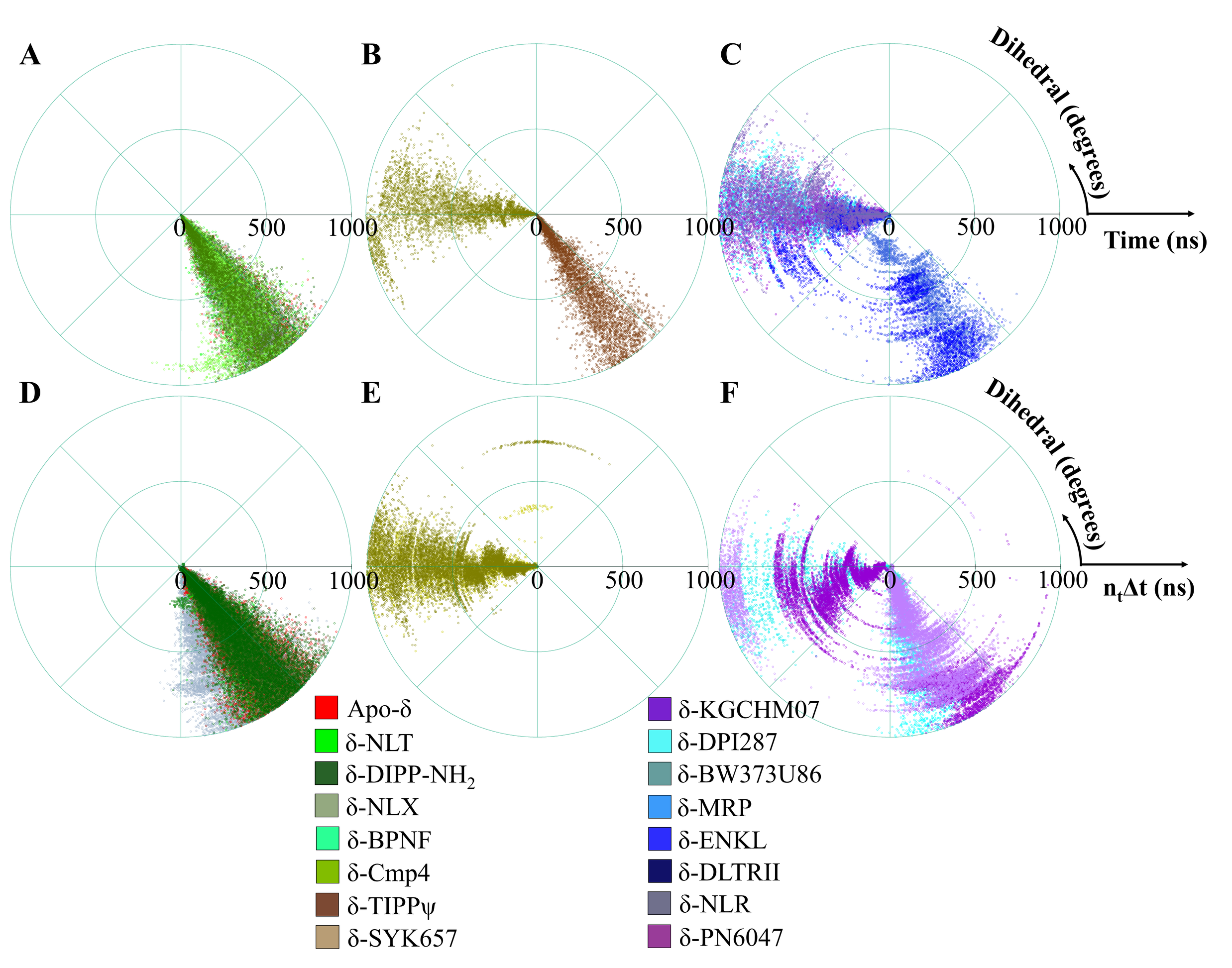

Supplement: S15 Fig — Polar plots for the cMD and GaMD simulations for A and D: Non-agonized systems, B and E: Inverse agonized systems, and C and F: Full agonized systems. The apo, as well as naltrindole, DIPP-NH2, nalorphine, naloxone, buprenorphine, and compound 4 complexes, are invariant in their rotamers, oriented towards the CCS. Between the inverse agonists, only the SYK657 complex adopts the non-agonized rotamer, while the TIPPψ complex resembles the full agonized systems, that predominantly possess the outward rotamer. In the cMD sampling, only morphine and enkephalin-L complexes fluctuate in the dihedral angle populations. In the GaMD sampling, one replicate of DPI287 adopts the outward orientation, and the other, the upward rotamer, while the two replicates of KGCHM07 complex adopt the two rotamers. (TIF) [file pone.0304068.s015.tif]

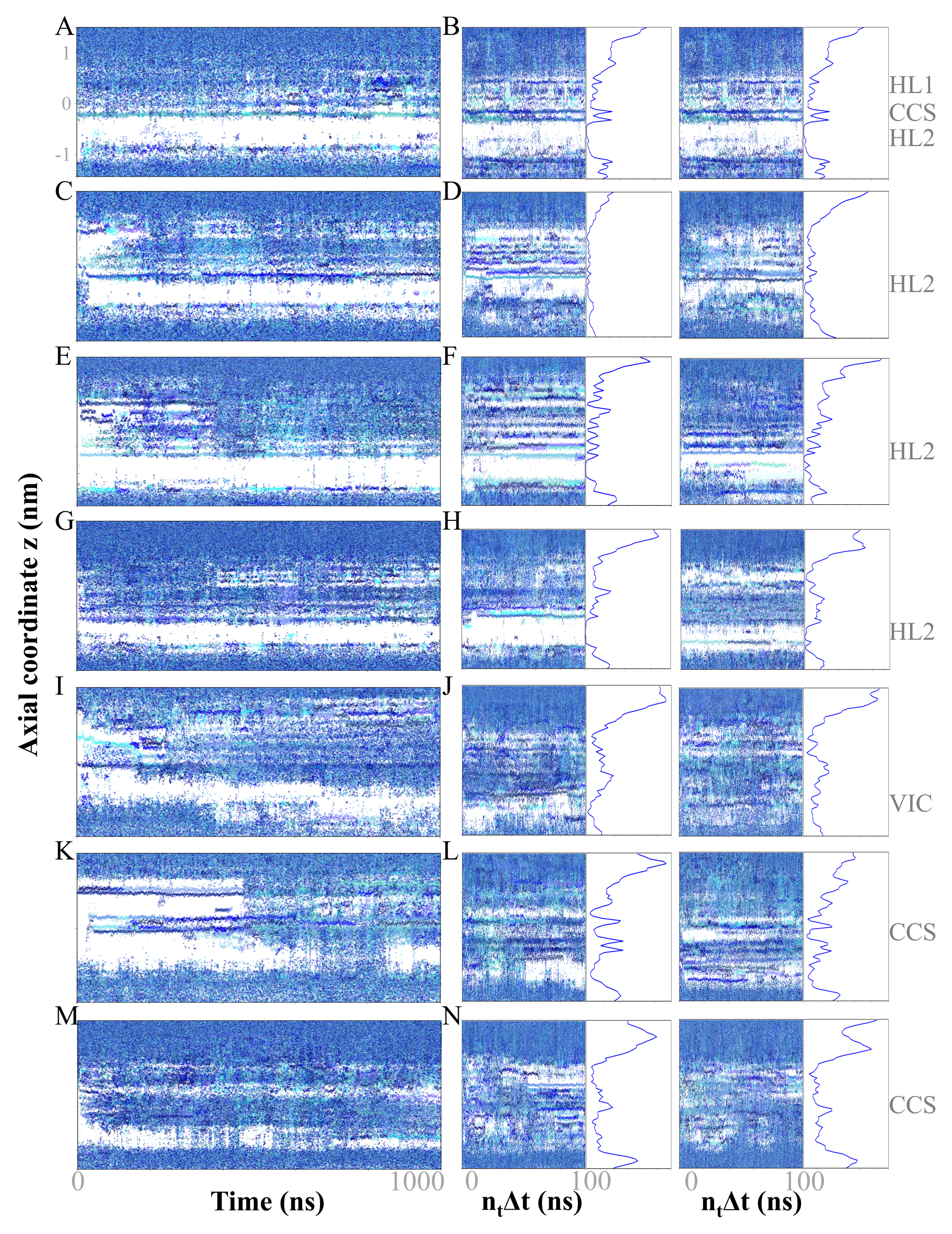

Supplement: S16 Fig — The tendencies of hydration observed in the cMD sampling continue in our accelerated simulations. For (A) apo-δ, (C) δ-NLT, (E) δ-DIPP-NH2, and (G) δ-NLR, the water molecules do not penetrate notably into the hydrophobic layer 2 (HL2), where Y3187.53 is located. During the GaMD replicates, (B, D, F, and H respectively), HL2 remains with low water molecule interactions. Nevertheless, the main agonized systems, (I) δ-KGCHM07, (K) δ-DPI287 and (M) δ-TIPPψ, that begin the HL2 hydration during our cMD simulations, fully hydrate the interhelix pore during the GaMD sampling (J, L, and N respectively). (TIF) [file pone.0304068.s016.tif]

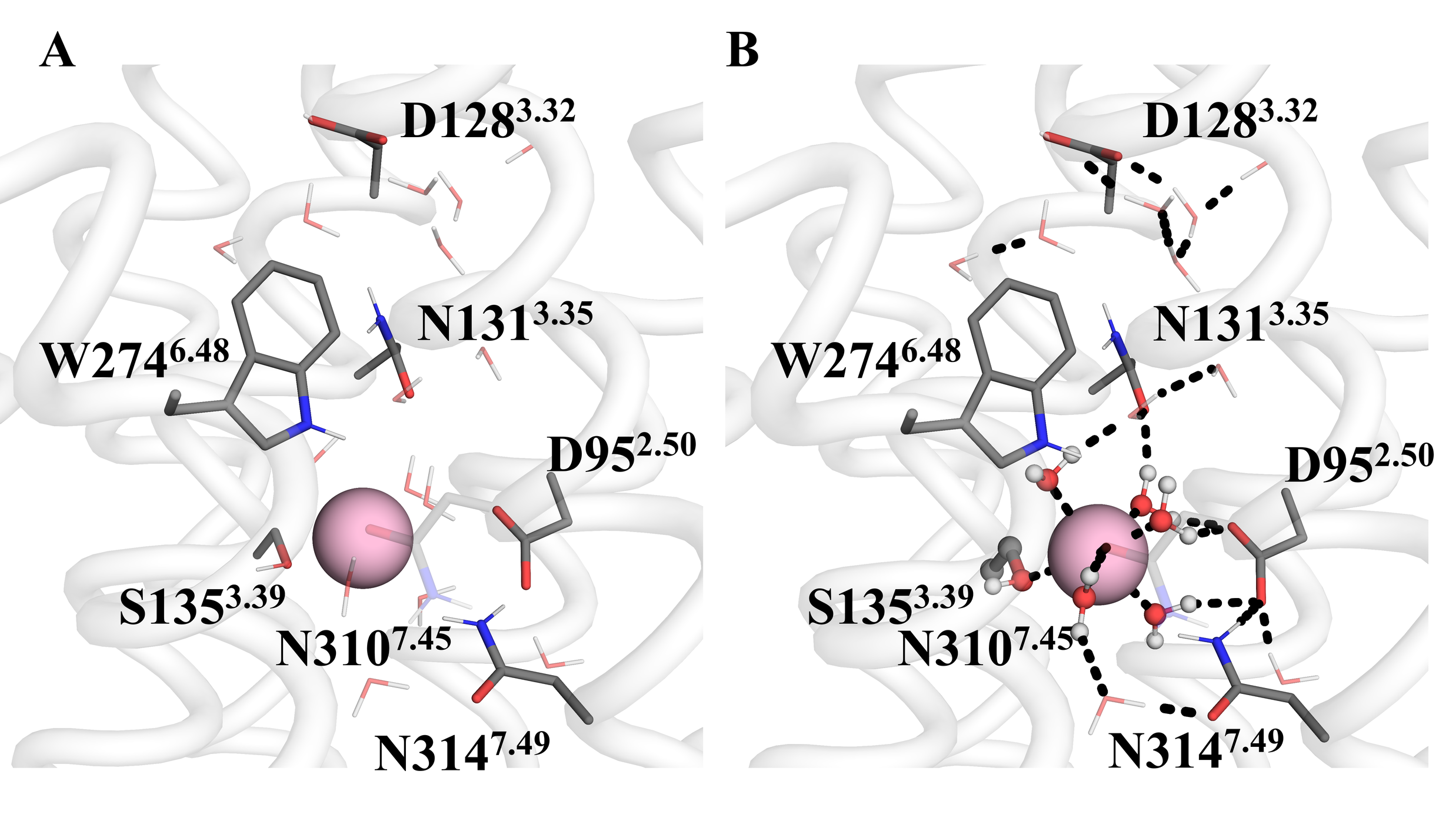

Supplement: S17 Fig — The cation is surrounded by a water molecule network that also interact with the transmission switch W2746.48 and N1313.35. (TIF) [file pone.0304068.s017.tif]

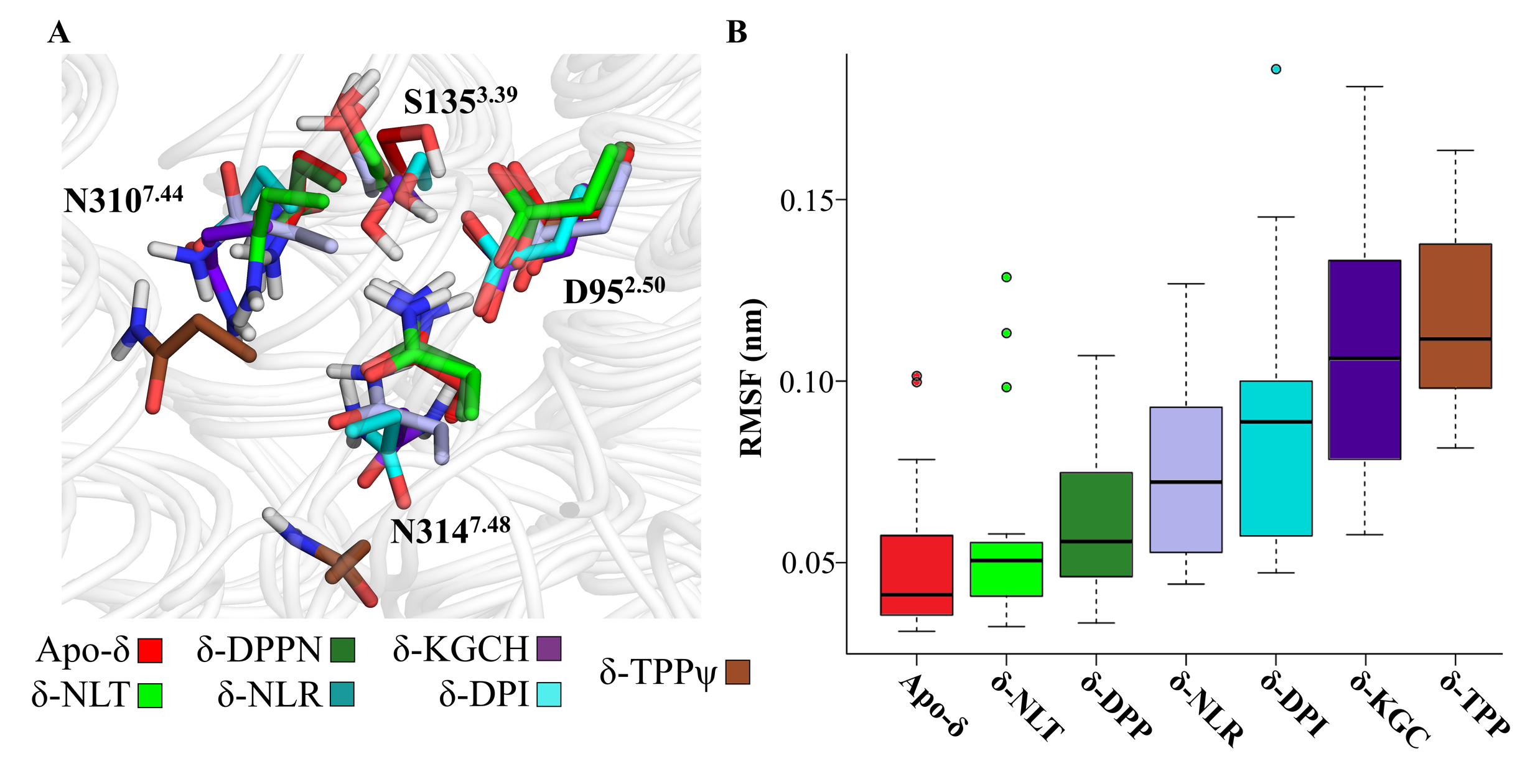

Supplement: S18 Fig — The residues D952.50, S1353.39, N3107.45 and N3147.49 form the central sodium ion pocket. (A) Median configuration of the CCS. The Apo, naltrindole, DIPP-NH2 and nalorphine are similar in the representative configuration, whereas in the TIPPψ the CCS is collapsed due the unfolding and displacement of TM7. (B) The RMSF of the sidechain of those residues (along with C2736.47) is lower in the Na+-coordinated and antagonized systems, and greater in the agonized systems, with the inverse agonist TIPPψ complex is the highest. (TIF) [file pone.0304068.s018.tif]

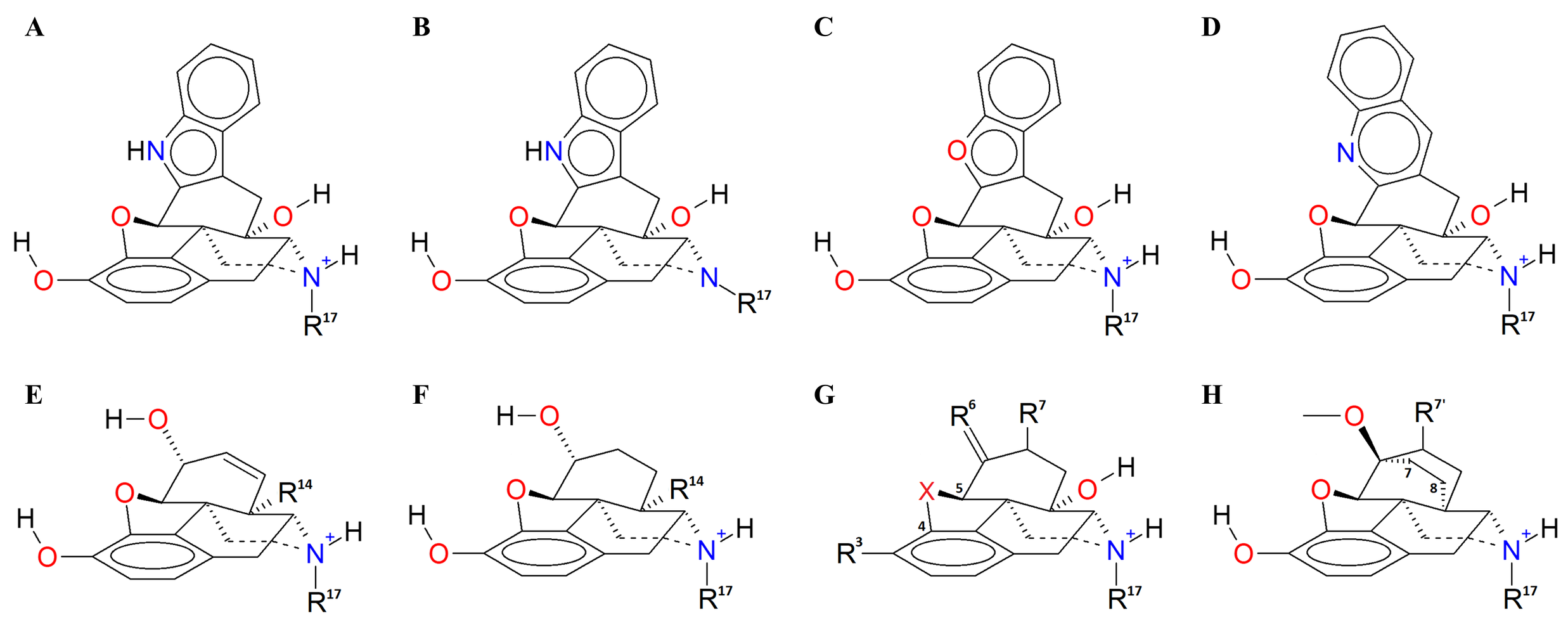

Supplement: S19 Fig — (A) Morphindole, protonated, (B) morphindole, non-protonated, (C) morphobenzofuran (protonated), (D) morphoquinoline, (E) 7,8-dehydro-4,5-epoxymorphinan, (F) 4,5-epoxymorphinan, (G) 6-(methylene/ oxo)morphinan, and (H) orvinol core. The shift of N17-methyl to N17-allyl in the mild-agonist morphine, originates the partial agonist nalorphine; and from the partial agonist oxymorphone to the antagonist naloxone. Also, the shift from N17-cyclopropylmethyl to N17-cyclobutylmethyl, from the agonist 6β-naltrexol to the partial agonist nalbuphine [95]; implying this change a voluminous substituent than those of the morphinan antagonists. In agreement with this facts, it was reported [33] than electron-withdrawing groups as N17-substituents in the DOR antagonists are important for its activity. The shifting of the cyclopropylmethyl group in naltrindole with phenylacetyl produces the full agonist SYK754, and its replacement with benzylsulfonyl and phenethylsulfonyl substituents, the corresponding partial agonists are produced (47.1% and 88.1% of efficacy, respectively, with respect the reference agonist DPDPE). Secondly, the 17-bencenesulfonyl derivative possesses antagonistic activity, while the mesylyl (CH3SO2-), triflyl (CF3SO2-), cyclopropylsulfonyl, and vinylsulfonyl substitutions generate partial inverse agonists (-48.8%, -36.1%, -80.5% and -80.2% respectively). And finally, the cyclopropylcarbonyl (SYK623) derivative is a near-full inverse agonist (up to -69% of efficacy with respect SNC80 [121]) Regarding the differences between carboxamides and sulfonamides, it is reported [122] that the former are chemically harder and with lesser dipole moment than the latter. Also, the phenylsulfonyl moiety increases the basicity in both heteroatoms, higher in the N atom and lesser in the O atoms, whereas the trifluoromethanosulfonyl group decreases the basicity over the same atoms, both with respect to the methanosulfonyl substituent. It is more suitable that the N atom in sulfona [file pone.0304068.s019.tif]
