# Supplementary material for: Descriptive molecular pharmacology of the δ opioid receptor (DOR): A computational study with structural approach
Source: PLoS One. 2024 Jul 11;19(7):e0304068. doi: 10.1371/journal.pone.0304068 (PMC11239112; doi:10.1371/journal.pone.0304068)
Supplement: S1 Table — a. We conducted three replicates of the naloxone complex to assess a representative conformational ensemble. (DOCX) [file pone.0304068.s020.docx]

| **System** | **Ligand name** | **Ligand structural class** | **Ligand activity** | **PDB ID template** |
| --- | --- | --- | --- | --- |
| δ-NLX^a^ | Naloxone | Morphinan | Non-selective, putative δ_2_-preferred antagonist | 4EJ4^12^, 4N6H^3^ |
| δ-BPNF | Buprenorphine | Orvinol (oripavine-derived, Bentley adduct, 6,14-endoethanomorphinan) | Multifunctional: δ, κ antagonist/μ partial agonist |  |
| δ-Cmp4 | Compound 4 | Cyclopeptide | Selective δ antagonist | 6PT2^13^, 8F7S[120] |
| δ-PN6 | PN6047 | Benzhydrylidenepiperidine | Biased δ agonist | 6PT3^13^ |
| δ-BW3 | BW373U86 | Benzhydrylpiperazine | Selective full δ agonist |  |
| δ-MRP | Morphine | Morphinan | Non-selective, near-full δ agonist | 4EJ4^12^, 4N6H^3^ |
| δ-ENKL | Enkephalin L | Peptide | Non-selective, δ_1_ full agonist/ δ_2_ inverse agonist | 6PT2^13^ |
| δ-DLTR | Deltorphin II | Peptide | Selective δ_2_ full agonist | 8F7S[120] |
| δ-SYK | SYK657 | Morphinan | Selective δ_2_ full inverse agonist | 4EJ4^12^, 4N6H^3^ |
