# Supplementary material for: Descriptive molecular pharmacology of the δ opioid receptor (DOR): A computational study with structural approach
Source: PLoS One. 2024 Jul 11;19(7):e0304068. doi: 10.1371/journal.pone.0304068 (PMC11239112; doi:10.1371/journal.pone.0304068)
Supplement: S2 Table — Structure of the ligands considered for the QSAR. Structure of (A) morphindoles: naltrindole and derived Hirayama ligand series (light-yellow shaded), (B) sulfonylmorphindoles of Iwamatsu series (light-green shaded), (C) morphobenzofurans: naltriben and its analogue SYK656, (D) morphoquinoline Nemoto series (light-blue shaded), (E) 4,5-epoxymorphinans: morphine, codeine, nalorphine and dehydronalbuphine, (F) 7,8-dihydro-4,5-epoxymorphinans, (G) morphinan-6-one relatives, and (H) dihydronororvinoles. (DOCX) [file pone.0304068.s021.docx]

| **Core A** | | | | **Core B** | | | **Core C** | | | | | | **Core D** | | | | |
| --- | --- | --- | --- | --- | --- | --- | --- | --- | --- | --- | --- | --- | --- | --- | --- | --- | --- |
| **R^17^** | | **Name** | | **R^17^** | **Name** | | **R^17^** | | | **Name** | | | **R^17^** | | | | **Name** |
| -CH_2_CH(CH_2_)_3_ | | Naltrindole | | -COCH(CH_2_)_3_ | SYK623 | | -CH_2_CH(CH_2_)_3_ | | | Benzofuran | | | -CH_2_CH(CH_2_)_3_ | | | | 1 |
| -CH=CH_2_ | | 1M | | -COCH_2_C_6_H_5_ | SYK754 | | -CH_2_C_6_H_5_ | | | SYK657 | | | -CH_2_CH_3_ | | | | 6A |
| -CH_2_CH_2_F | | 1C | | -SO_2_CH_3_ | 9A | |  | | | | | | -CH_2_CH_2_CH_3_ | | | | 6B |
| -CH_2_CHF_2_ | | 1B | | -SO_2_CF_3_ | 9B | |  |  |  |  |  |  | -CH_2_CH_2_CH_2_F | | | | 6C |
| -CH_2_CF_3_ | | 1A | | -SO_2_CH_2_C_6_H_5_ | 9C | | **Core H** | | | | | | | | | | |
| -CH_2_CCl_3_ | | 1F | | -SO_2_(CH_2_)_2_C_6_H_5_ | 9D | | **R^17^** | | | **R^7’^** | | | | | **7-8** | | **Name** |
| -CH_2_CH_2_CF_3_ | | 1D | | -SO_2_CH(CH_2_)_3_ | 9E | | -CH_3_ | | | -CH[OH][CH_3_](CH_2_)_2_CH_3_ | | | | | C=C | | Etorphine |
| -CH_2_CF_2_CF_3_ | | 1E | | -SO_2_CH=CH_2_ | 9G | | -CH_3_ | | |  |  |  |  |  | C‒C | | Dihydroetorphine |
| -C_6_H_5_ | | 1J | |  | | | -CH_2_CH(CH_2_)_3_ | | |  |  |  |  |  | C‒C | | RX6007M |
| -CH_2_C_6_H_5_ | | 1K | |  |  |  |  |  |  | -C[CH_3_]_2_[OH] | | | | | C‒C | | Diprenorphine |
| - CH_2_CH_2_C_6_H_5_ | | 1L | |  |  |  |  |  |  | -CH[OH][CH_3_]C(CH_3_)_3_ | | | | | C‒C | | Buprenorphine |
| **Core E** | | | | | | **Core G** | | | | | | | | | | | |
| **R^17^** | **R^14^** | | **R^3^** | **Name** | | **R^17^** | | **R^7^** | | | **R^6^** | **R^3^** | | **4-X-5** | | **Name** | |
| -CH_3_ | -H | | -H | Morphine | | CH_3_ | | -H,-H | | | O | -OH | | -O- | | Hydromorphone | |
| -CH_3_ | -H | | -CH_3_ | Codeine | | CH_3_ | | -H,-H | | | O | -OCH_3_ | | -O- | | Hydrocodone | |
| -CH=CH_2_ | -H | | -H | Nalorphine | | CH=CH_2_ | | -H,-H | | | O | -OH | | -O- | | Naloxone | |
| -CH_2_CH(CH_2_)_3_ | -OH | | -H | Dehydronalbuphine | | CH_2_CH(CH_2_)_2_ | | -H,-H | | | O | -OH | | -O- | | Naltrexone | |
| **Core F** | | | | | | CH_2_CH(CH_2_)_2_ | | =CHC_6_H_5_ | | | O | -OH | | -O- | | Benzylidenenaltrexone | |
| **R^17^** | **R^14^** | | **R^3^** | **Name** | | CH_2_C_6_H_5_ | | =CHC_6_H_5_ | | | O | -OH | | -O- | | SYK656 | |
| -CH_3_ | -H | | -H | Dihydromorphine | | CH_2_CH(CH_2_)_2_ | | -H,-H | | | CH_2_ | -OH | | -O- | | Nalmefen | |
| -CH_3_ | -H | | -CH_3_ | Dihydrocodeine | | CH_2_CH(CH_2_)_2_ | | -H,-H | | | O | -CONH_2_ | | -OH | | Samidorphan | |
| -CH=CH_2_ | -H | | -H | Dihydronalorphine | |  | | |  | | | | | | | | |
| -CH_2_CH(CH_2_)_3_ | -OH | | -H | Nalbuphine | |  |  |  |  |  |  |  |  |  |  |  |  |
