# Supplementary material for: Descriptive molecular pharmacology of the δ opioid receptor (DOR): A computational study with structural approach
Source: PLoS One. 2024 Jul 11;19(7):e0304068. doi: 10.1371/journal.pone.0304068 (PMC11239112; doi:10.1371/journal.pone.0304068)
Supplement: S3 Table — (DOCX) [file pone.0304068.s022.docx]

| **Bioactive molecule** | **Efficacy (%)** | **Gibbs energy (**×10^9^ **J/kg)** | **Ground state energy (×10^9^ J/kg)** | **Bioactive molecule** | **Efficacy (%)** | **Gibbs energy (**×10^9^ **J/kg)** | **Ground state energy (**×10^9^ **J/kg)** |
| --- | --- | --- | --- | --- | --- | --- | --- |
| SYK656 | -103 | -8.533 | -8.535 | SMDF (samidorphan) | 21 | -8.676 | -8.678 |
| SYK657 | -99 | -8.569 | -8.572 | OXCD (oxycodone) | 21 | -8.757 | -8.759 |
| SYK623 | -91.2 | -8.678 | -8.681 | HY1e | 21.1 | -9.582 | -9.584 |
| IW9f | -80.5 | -10.47 | -10.47 | NM6c | 27.1 | -8.779 | -8.782 |
| IW9g | -80.2 | -10.56 | -10.57 | HMRO (hydromorphone) | 29 | -8.624 | -8.627 |
| HY1k | -56.5 | -8.472 | -8.475 | NLBF (nalbuphine) | 30 | -8.593 | -8.596 |
| IW9a | -48.8 | -10.63 | -10.63 | COD (codeine) | 31 | -8.565 | -8.568 |
| HY1b | -48.6 | -9.033 | -9.036 | NM1 | 32.4 | -8.480 | -8.483 |
| HY7a | -48.5 | -9.290 | -9.293 | OXMR (oxymorphone) | 33 | -8.821 | -8.824 |
| HY1a | -44.8 | -9.255 | -9.257 | HCDO (hydrocodone) | 39 | -8.565 | -8.568 |
| HY1c | -38.1 | -8.792 | -8.795 | HY1l | 40.3 | -8.439 | -8.442 |
| IW9b | -36.1 | -10.105 | -11.05 | NM6a | 41.2 | -8.524 | -8.526 |
| BNTX | -10.2 | -8.549 | -8.552 | MRP (morphine) | 43 | -8.624 | -8.627 |
| NTB (naltriben) | -1.6 | -8.589 | -8.592 | IW9d | 47.1 | -10.23 | -10.24 |
| HY1j | 4.1 | -8.525 | -8.527 | DPNF (diprenorphine) | 55 | -8.424 | -8.427 |
| NLT (naltrindole) | 7.5 | -8.484 | -8.487 | NLR (nalorphine) | 58 | -8.556 | -8.559 |
| NLX (naloxone) | 8 | -8.741 | -8.744 | NM6b | 62.4 | -8.484 | -8.487 |
| HY1f | 12.2 | -16.44 | -16.44 | NLMF (nalmefen) | 66.5 | -8.458 | -8.461 |
| HY1m | 16.2 | -8.523 | -8.526 | HDC (hydrocodeine) | 69 | -8.519 | -8.522 |
| BPNF (buprenorphine) | 19 | -8.329 | -8.332 | SRI22141 | 73 | -10.41 | -10.41 |
| HY1d | 19.3 | -9.197 | -9.199 | HMRP (hydromorphine) | 76 | -8.575 | -8.578 |
| NTX (naltrexone) | 25 | -8.383 | -8.688 | IW9e | 88.1 | -10.16 | -10.16 |
|  |  |  |  | ETRF (etorphine) | 107 | -8.460 | -8.463 |
