# Supplementary material for: Descriptive molecular pharmacology of the δ opioid receptor (DOR): A computational study with structural approach
Source: PLoS One. 2024 Jul 11;19(7):e0304068. doi: 10.1371/journal.pone.0304068 (PMC11239112; doi:10.1371/journal.pone.0304068)
Supplement: S4 Table — Note: *** stands for high significance, ** for mild significance, and * for low significance. (DOCX) [file pone.0304068.s023.docx]

| **Variable** | **Coefficient** | **Standard error** | **Calculated t-Student** | **p value** |
| --- | --- | --- | --- | --- |
| G | 3.731×10^-2^ | 1.013×10^-2^ | -3.683 | 0.001017** |
| G^2^ | -1.171×10^-2^ | 2.885×10^-3^ | -4.060 | 0.000377*** |
| G^3^ | -9.545×10^-4^ | 2.622×10^-4^ | -3.640 | 0.001138** |
| GE_0_ | 2.508×10^-3^ | 6.830×10^-4^ | 3.671 | 0.000328** |
| GE_0_^3^ | 8.939×10^-6^ | 3.126×10^-6^ | 2.859 | 0.008089** |
| G^3^E_0_^3^ | -3.180×10^-8^ | 1.163×10^-8^ | -2.734 | 0.010905* |
| E_0_ | 3.836×10^-2^ | 1.030×10^-2^ | 3.725 | 0.000913*** |
| E_0_^2^ | 9.681×10^-3^ | 2.918×10^-3^ | 3.318 | 0.002599** |
| E_0_^3^ | 1.055×10^-3^ | 2.774×10^-4^ | 3.804 | 0.000741*** |
| β_0_ (intercept) | 2.391×10^6^ | 7.561×10^5^ | 3.163 | 0.003840** |
