# Supplementary material for: Descriptive molecular pharmacology of the δ opioid receptor (DOR): A computational study with structural approach
Source: PLoS One. 2024 Jul 11;19(7):e0304068. doi: 10.1371/journal.pone.0304068 (PMC11239112; doi:10.1371/journal.pone.0304068)
Supplement: S5 Table — (DOCX) [file pone.0304068.s024.docx]

| **Residual standard error** | RSE = 40.36 |
| --- | --- |
| **Determination coefficient** | r^2^ = 0.6084 |
| **Adjusted determination coefficient** | r*_adj_*^2^ = 0.4779 |
| **Calculated Fisher value for regression** | F_c_ = 4.662 |
| **Degrees of freedom of regression** | ν_1_ = 9, ν_2_ = 27 |
| **p-value of regression** | 0.0008682 |
| **Pearson correlation coefficient** | r*_obs;calc_* = 0.771*** |
